# Measuring sexual behaviour in Malawi: a triangulation of three data collection instruments

**DOI:** 10.1186/s12889-018-5717-x

**Published:** 2018-06-28

**Authors:** Nicola Desmond, Nico Nagelkerke, Wezzie Lora, Effie Chipeta, Mwiza Sambo, Moses Kumwenda, Elizabeth L. Corbett, Miriam Taegtemeyer, Janet Seeley, David G. Lalloo, Sally Theobald

**Affiliations:** 1 Liverpool School of Tropical Medicine, Pembroke Place, Liverpool, UK; 2 Malawi-Liverpool-Wellcome Trust Clinical Research Programme, Blantyre, Malawi; 3 College of Medicine, University of Malawi, Blantyre, Malawi; 4 London School of Hygiene and Tropical Medicine, Keppel Street, London, UK

**Keywords:** Sexual behaviour, Measurement, HIV self-testing, ACASI, Coital diaries, Malawi

## Abstract

**Background:**

There is a need for valid approaches to measure sexual interactions to assess the impact of behavioural interventions and to predict the impact of behaviour changes. Different methods of asking about sexual behaviour often yield conflicting answers and men often report higher levels of heterosexual activity than women. To better understand self-reported sexual behaviour data and how best to collect it, we analyzed data collected as part of a larger project (ST IMPACTS) on the social and behavioural impact of introducing community-level HIV self-testing (HIVST) with counseling (semi-supervised with pre- and generic post-test counseling provided on delivery or collection of test kits) in an urban Malawian setting.

**Methods:**

Information on sexual behaviour was collected from HIV self-testers over a three-month period. Three different methods were used: retrospective face-to-face interviews (FTFI); audio computer assisted self-interviews (ACASI) and a prospective coital diary. Both retrospective instruments were used before and after the three-month study period. Frequency and cross-tabulation, as well as scatterplots, were used for exploratory analyses. Chi-square tests were used to test for differences in proportions. Spearman’s correlation coefficient was used to explore associations between both continuous and ordinal variables and Wilcoxon’s paired sample and Mann-Whitney test was used to test for differences in such variables or between variables.

**Results:**

There was reasonable agreement between the two retrospective methods although both yielded inconsistent answers e.g. with lower reported numbers of life-time sexual partners at the end than at the beginning of the study period. The diary method elicited higher reported levels of sex with multiple partners than both retrospective instruments which may be due to inadequate recall. Over the study period 37.4% of men and 19.7% of women reported multiple sexual partners using the diary. There was no clear relationship between reported sexual behaviour and HIV status (prevalence 9.6%).

**Conclusions:**

Diaries may therefore have higher validity for sensitive behaviour reporting and thus be the preferred method in similar African contexts in measuring sexual behaviours.

**Electronic supplementary material:**

The online version of this article (10.1186/s12889-018-5717-x) contains supplementary material, which is available to authorized users.

## Background

In several severely affected countries in Sub-Saharan Africa (SSA), HIV prevalence and incidence appear to be declining. Nevertheless, HIV morbidity and mortality are still having devastating effects on affected populations [1]. Although the roll-out of Antiretroviral Therapy (ART) during the last decade has dramatically reduced HIV-related morbidity and mortality, our ultimate goal should still be elimination of the virus, at least as a generalized epidemic [2] which requires substantial reductions in transmission [3]. Moves towards a universal test and treat approach (UTT) are underway, and as it is estimated that between 30 and 40% of adults in HIV-endemic resource constrained settings have had an HIV test [4]; this may be expected to considerably increase ART use with consequent reductions in HIV infectiousness. Still, difficulties in identifying all HIV infected individuals and maintaining them on treatment suggest that without behaviour change, the prospects of elimination may be limited [5]. There is an increase in the number of interventions that target uninfected individuals, such as Pre-Exposure Prophylaxis (PrEP), and microbicidal vaginal gels; but limited availability and poor adherence limit their effectiveness [6].

An important goal of HIV prevention remains reducing sexual risk behaviours in both HIV-infected and uninfected individuals. Both the number of sexual contacts between HIV positive and negative individuals, and behaviours that affect the risk of transmission during such contacts, such as (consistent) condom use, ART use, or male circumcision determine this risk [7]. In couples, knowledge of each other’s sero-status may be an essential element in reducing transmission [8]. Behaviours that affect the connectedness of the sexual network – a key determinant of the epidemic spread of sexually transmitted infections – include having multiple partners within a short time-span, either simultaneously (“concurrent”) or in quick succession [9]. There is a need for valid approaches to monitor and measure sexual interactions in order to assess the impact of behavioural interventions and to predict the impact of behaviour changes - either as the consequence of interventions, autonomous changes, or because of selection for lower risk behaviour. Sexual behaviour however, is a private activity, surrounded by gender norms, societal proscriptions and prescriptions, and notoriously challenging to measure. While asking people about their sexual behaviour may sound easy in principle, it can be difficult in practice. Different methods of asking about sexual behaviour often yield conflicting answers and men often report higher levels of heterosexual activity, such as numbers of partners or number of sexual acts with a partner, than women, which is improbable [10]. It has been suggested that this is mostly due to underreporting by women rather than over-reporting by men [11]. Another methodological problem is that the validity of instruments, e.g. personal interviews or anonymous questionnaires, may depend on the context, culture, norms and understanding of the study population. Validation of instruments should therefore, preferably, be replicated in each study population. Biomarkers of sexual activity exist, at least for women, but these are either too complex or costly for large scale use, and/or ethically controversial, for example through identifying issues such as non-paternity or half-sibling testing [12, 13]. It is therefore unlikely that methods of self-reporting will soon be replaced by more “biological” methods.

To understand better self-reported sexual data and how best to collect it, we analyzed data from a larger project (ST IMPACTS) on the social and behavioural impact of introducing community-level HIV self-testing (HIVST) with counseling (semi-supervised with pre and generic post-test counseling provided on delivery or collection of test kits) in an urban Malawian setting. The parent project focused specifically on gender-based violence, sexual risk taking, and risk-compensation in the context of self-testing. In this sub-study, we estimate levels of multiple partner sex over a three-month period, using three different instruments: an Audio Computer Assisted Self Interview (ACASI), a Face to Face Interview (FTFI) with a specially trained field worker, both retrospective over the past 3 months, and a prospective self-completed pictorial diary. By comparing results from these methods, we aim to obtain better information on sexual behaviours relevant for HIV transmission and validate these instruments for this setting. The best instrument could then be further validated for its suitability to monitor behaviour change within urban Malawian and other contexts.

## Methods

### Sub-study participants

**Fig. 1:**
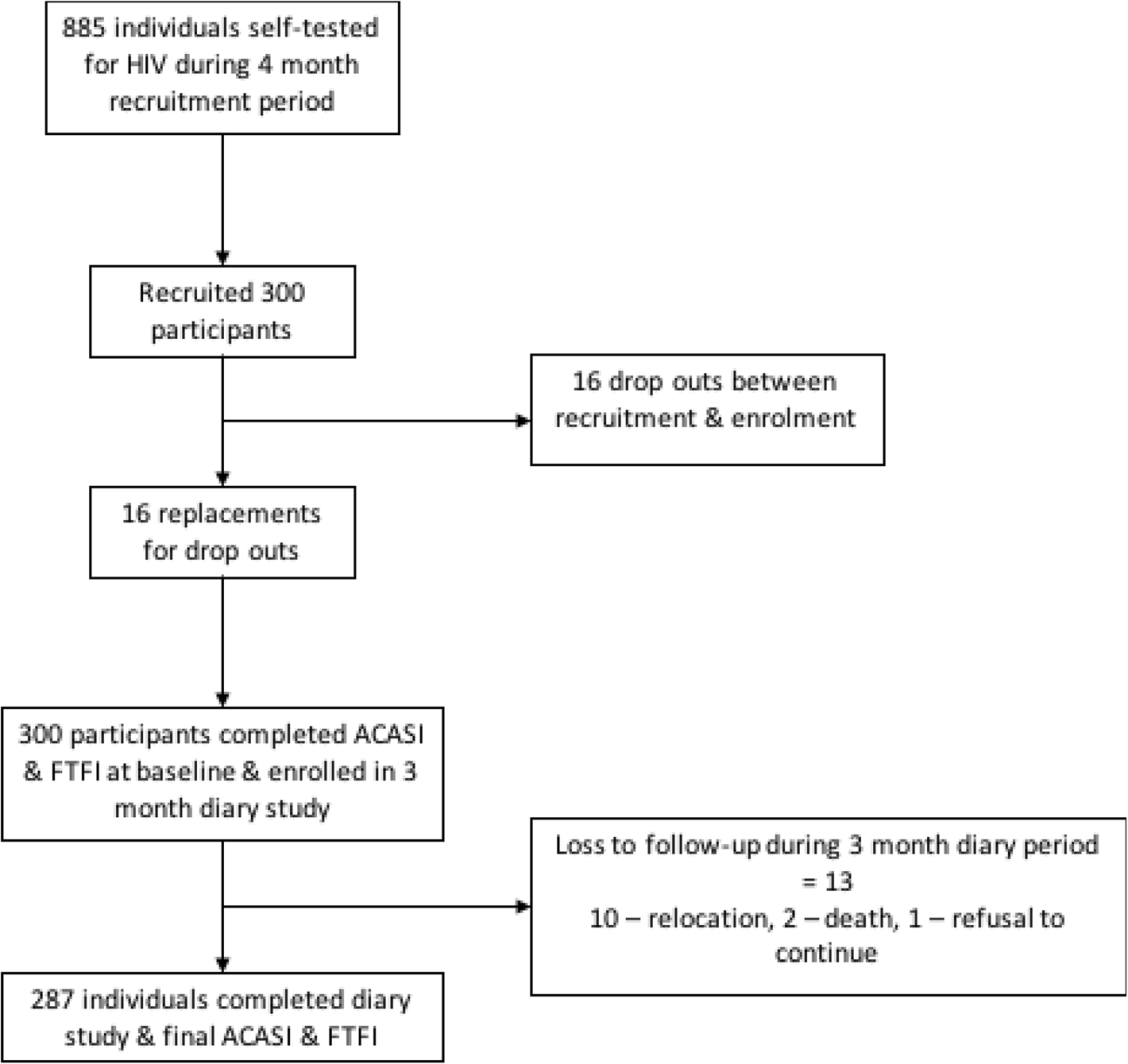
Enrolment and attrition flowchart

During a four-month recruitment period between October 2014 and January 2015, participants between (16–49 years of age) of an HIV self-testing intervention (HIVST) were recruited in an area covered by the Hit TB project [14]. During these months 885 people had access to an HIVST kit. Of these 316 were re-contacted at home by counselors to discuss whether they would participate in our (sub) study on sexual behaviour and gender-based violence. Among these 16 dropped out between initial verbal agreement to participate and formal enrolment in the study and were replaced. All individuals who accepted HIV self-test kits were eligible, whether or not they actually tested following the acceptance of the kits. We finally recruited 300 participants (see Fig. 1). Participants included both individuals and couples normally resident in poor, high density, areas of urban Blantyre, the second largest city of Malawi with high levels of in- and out migration. Confirmatory HIV testing and onward referral were offered to all participants. The study was approved by the College of Medicine Research Ethics Committee in Malawi and the Liverpool School of Tropical Medicine Ethics Committee in the UK (P.02/13/1341). Nic, the numbers appear inconsistent. If the 16 were replaced thn the number recruited are 316 not 300. Please check.

### Data collection methods

**Fig. 2:**
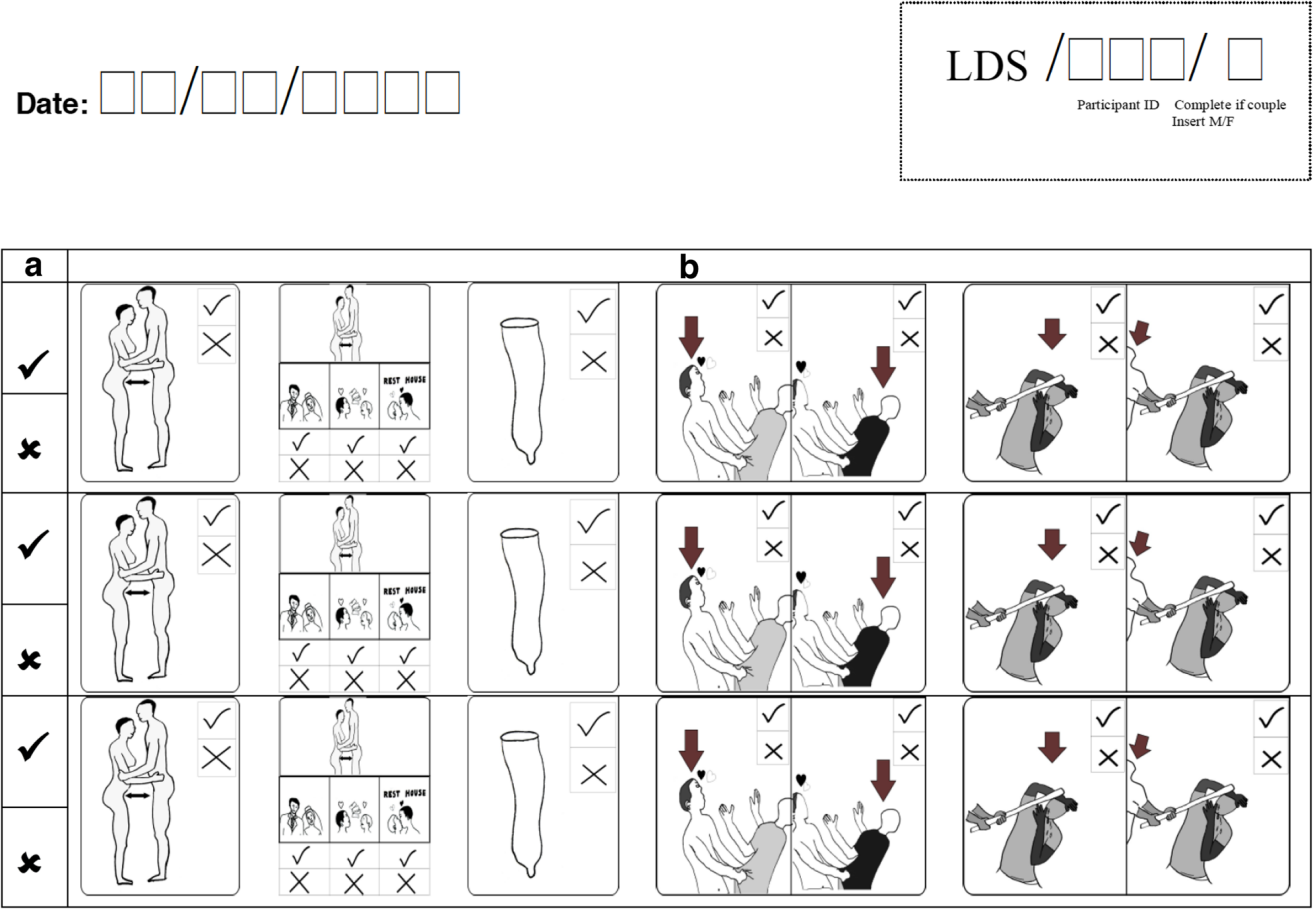
Pictorial diary

Three widely used data collection methods for assessing sexual behaviour were compared: ACASI, FTFI, and a 3-month longitudinal pictorial diary Fig. 2. The diary tool was developed through a collaborative and iterative process with an artist and community groups to optimize comprehension and acceptability. Following a short pilot study to assess whether the instruments were understood and testing feasibility of the study, and following information sharing and written consent, all 300 participants were requested to complete a short enrolment questionnaire using ACASI with both oral and visual presentation of questions to address literacy issues [15–18]. In all cases this was followed later that day by an FTFI carried out by field workers gender matched to the participant using a questionnaire with visual recall aides. Information was elicited consistently across FTFI and ACASI on socio-demographic variables such as age, sex, marital status, surviving children, and also on previous HIV testing behaviour, disclosure of HIV results to partners, any incidents of gender-based violence (GBV), coercive testing, and history of sexual behaviour (cf. online Additional file 1). Sexual behaviour questions asked about life-time sexual partners, sexual partners in the past 3 months, and type of partner, e.g. spousal, regular or irregular. Participants were then asked to complete a daily pictorial diary for a three-month study period (each diary covered a period of 2 weeks with a total of 6 diaries per participant for the 12 week diary study period). Those who agreed received full training before taking the diary home. This diary included data on, sexual behaviour, household dynamics, disclosure and incidents of gender-based violence as well as coercive testing. Sexual behaviour questions elicited details about each sexual intercourse, the type of partner (spousal/cohabiting, other regular, irregular partners) and if a condom was used. Diary completion was monitored and supported through regular, formal visits every 2 weeks to collect the diary and informal visits on an ad hoc basis to participants to address any concerns and ensure that the diary was being completed daily [19–21]. At the end of the three-month data collection period, a second ACASI and FTFI were completed using these 3 months as the recall period. Data on the three sources of reported behaviour were then compared and triangulated.

### Statistical methods

Sample sizes were calculated to have 80% power to detect a correlation coefficient of 0.20, at the 5% significance level. Sample sizes were then increased by 50% to allow for subset analyses. Individuals (28) with < 30 diaries were excluded from analysis to ensure consistency of time periods in comparisons. Frequency and cross-tabulation, as well as scatterplots, were used for exploratory analyses. Chi-square tests were used to test for differences in proportions. Continuous and ordinal variables were generally reported as median and interquartile range (IQR). Spearman’s correlation coefficient was used to explore associations between such variables and Wilcoxon’s paired sample and Mann-Whitney test to test for differences in such variables or between variables.

## Results

After exclusion of those participants with < 30 days of completed diaries representing the,^1^ we had a total of 287 participants (including 48 couples) available for analysis.

### HIV status

HIV prevalence in our cohort was close to the 10% national adult (15–49 years) sero-positivity prevalence reported by UNAIDS but somewhat less than the 2010 DHS prevalence estimate of 14.5% for the Southern region of Malawi that includes the city of Blantyre (http://www.unaids.org/en/regionscountries/countries/malawi, http://dhsprogram.com/pubs/pdf/HF34/HF34.pdf). 16/144 of female and 11/136 of male participants who were tested on enrolment to the study were HIV positive. HIV positive participants were on average 1.86 years older than negative participants but this was not statistically significant. Among 48 couples included, 8 were affected by HIV, 4 discordant with 3 with an HIV positive male partner.

### Retrospective instruments FTFI and ACASI

#### Reported demographic and personal data

##### Age

Participants (by ACASI) had a median age of 26 years in both women (IQR: 22–31) and men (IQR: 24–33).

##### Religion

In the baseline FTFI, 93% of participants reported to be Christians. With one exception (atheist), all others reported to be Muslims. Religion was not elicited in the ACASI.

##### Marital status

Marital status was classified in baseline FTFI. 78/139 (56.1%) men and 108/147 (73.5%) women reported being currently married or cohabiting. 51 (83.6%) unmarried men and 19 (48.7%) unmarried women reported that they had never been married. Table 1 shows the marital status at the two time points reported by each of the two retrospective instruments. Marital status reported by ACASI and FTFI did not fully agree. For example, three participants “unmarried” by FTFI reported to be married/cohabiting through the ACASI method. For one participant, the difference was the other way around. We do not know what has caused these differences between the two instruments.

**Table 1:** Self-reported marital status and sexual behaviour, both at baseline (pre) and after 3 months (post)

Variable	Sex			
	F (%)	M (%)	F (%)	M (%)
	Baseline (t0)		After 3 months	
ACASI				
Marital Status				
NEVER MARRIED	19 (12.9)	51 (36.7)	13 (9.5)	51 (38.1)
MARRIED OR LIVING WITH PARTNER	103 (70.1)	70 (50.4)	96 (70.6)	65 (48.5)
REMARRIED AFTER DIVORCE/DEATH	5 (3.4)	8 (5.8)	14 (10.3)	7 (5.2)
DIVORCED/SEPARATED	16 (10.9)	9 (6.5)	10 (7.4)	10 (7.5)
WIDOWED	4 (2.7)	1 (0.6)	3 (2.2)	1 (0.7)
Condom Use Last Sex				
Yes	29 (19.7)	42 (30.9)	24 (17.6)	53 (39.6)
No	118 (80.3)	94 (69.1)	112 (82.4)	81 (60.4)
Life Time Sex Partners				
None	5 (3.4)	5 (3.6)	2 (1.5)	2 (1.5)
1	41 (27.9)	21 (15.1)	45 (33.1)	19 (14.3)
3–5	96 (65.3)	91 (65.5)	83 (61.0)	92 (69.2)
6+	5 (3.4)	22 (15.8)	6 (4.4)	20 (4.4)
3 Months Sex partners				
None	21 (14.3)	23 (16.5)	16 (16.5)	21 (15.7)
1	121 (82.3)	98 (70.5)	116 (70.5)	96 (71.6)
3–5	3 (2.0)	17 (12.3)	1 (12.3)	16 (11.9)
6+	2 (1.4)	1 (0.7)	3 (0.7)	1 (0.8)
FTFI				
Marital Status				
NEVER MARRIED	17 (11.6)	50 (36.0)	13 (8.8)	51 (38.3)
MARRIED OR LIVING WITH PARTNER	104 (70.7)	68 (49.0)	103 (70.1)	65 (48.9)
REMARRIED AFTER DIVORCE/DEATH	11 (7.5)	7 (5.0)	13 (12.9)	9 (6.8)
DIVORCED/SEPARATED	10 (6.8)	13 (7.4)	8 (5.4)	8 (6.0)
WIDOWED	5 (3.4)	1 (0.6)	4 (2.8)	0 (0.0)
Condom Use Last Sex				
YES	30 (20.4)	42 (30.2)	29 (20.6)	55 (41.4)
NO	117 (79.6)	97 (69.8)	112 (79.4)	78 (58.6)
Life Time Sex Partners				
None	5 (3.4)	7 (5.0)	2 (1.4)	3 (2.3)
1	43 (29.3)	19 (13.7)	48 (34.0)	16 (12.0)
3–5	92 (62.6)	90 (64.7)	83 (57.9)	93 (69.9)
6+	7 (4.7)	23 (16.5)	8 (5.7)	21 (15.8)
3 Months Sex partners				
None	24 (16.3)	31 (22.3)	20 (14.2)	20 (15.0)
1	113 (76.9)	88 (63.3)	115 (81.6)	85 (63.9)
3–5	8 (5.4)	18 (13.0)	4 (2.8)	27 (20.3)
6+	2 (1.4)	2 (1.4)	2 (1.4)	1 (0.8)

As expected there was some fluidity in household arrangements. After 3 months, five participants reported having separated (FTFI), while nine participants had reportedly entered into a cohabiting arrangement. The same numbers were reported through ACASI.

20 (13.7%) of the women and 54 (38.8) of the men reported having no children through both the ACASI and FTFI method. The mean reported number of living children was 2.19/2.20 (FTFI/ACASI) for women and 1.43/1.51 for men.

#### Reported data on sexual behaviours

##### Numbers of sexual partners

Table 1 presents the key sexual behaviour parameters at each of the two time points (baseline and after 3 months) reported through the two retrospective instruments. As a measure of current and recent sexual activity, we looked at the self-reported number of partners in the past 3 months. We analyzed results from the post 3 months FTFI and ACASI as their data can be directly compared to the diaries for this period. Through the FTFI, women and men reported a mean number of sex partners during the last 3 months of 1.24 and 1.15 respectively (ns). Means calculated from the ACASI were 1.17 and 1.20 (*p* = 0.014 by Mann-Whitney test) for women and men respectively.

Over the three-month period 4.2% of women and 21.1% of men reported more than one sexual partner by ACASI. For FTFI these proportions were 2.9 and 12.7% respectively. These percentages were both lower than those reported by the diary method. There was a positive association between the number of reported partners in the past 3 months between the two retrospective methods of ACASI and FTFI. The Spearman’s correlation coefficient between the two measures was 0.80, and there was no statistically significant difference between the two methods (ACASI and FTFI) in the numbers of 3-months partners by Wilcoxon’s paired samples test.

##### Life-time sex partners and rate of acquisition of new partners

By ACASI, 5 of the women (one of them HIV-positive) and 7 of the men reported never having had sex at baseline which declined to 2 and 3 respectively after 3 months. Through the FTFI, 5 women (3 being the same as through the ACASI) reported never having had sex at baseline, again going down to 2 after the 3 month period. Only 5 men by FTFI and 6 by ACASI, declining to 2 and 3 after 3 months respectively (1 in each reporting being sexually experienced at baseline!), reported never having had sex. One in 4 participants reported more life-time sex partners at baseline than after our 3-month diary period in both FTFI and ACASI. These kinds of inconsistencies in response have also been observed in other contexts (e.g. Uganda), and thus do not seem to be confined to this Malawian population [22]. We therefore considered it not possible to reliably estimate the rate of acquisition of new partners from the increase in reported life-time sex partners or to reliably estimate the number of life time sexual partners.

#### Transactional sex

Participants were not asked explicitly whether they practiced transactional sex. In many African cultures, including within Malawi, gifts, often some money, (from men to women) are an accepted – even desirable - practice and do not have the same association with sex work as it perhaps would in Western cultures [23, 24]. Many sexual partners, however, suggests that money may have been an important motivation for engaging in sex. Two women (one HIV+) reported (both by ACASI and FTFI) “twenty” (perhaps meaning many) partners in the past 3 months. Although having many partners may be under-reported, it does suggest that either in this area “professional” sex work may not be as common as it is in some other African cities or that sex workers were underrepresented in our sample. Only one woman voluntarily disclosed being a sex worker at the time of the study.

### Coital diaries

Table 2 presents several key sexual behaviour parameters reported by 3-month coital diaries. Women reported a median of 45.5 (IQR: 16–89) sex acts over the 3 month diary period, while men reported a median number of 31 (IQR: 10–70). Married participants reported a median of 51.5 (IQR:25.75–90.0) while unmarried (never married, widowed, divorced) participants reported a median of 10 sex acts (IQR: 0–40.5). There was a large gender discrepancy in the reported number of sex acts with cohabiting partners. While women (including those reporting no sex with a cohabiting partner) reported a median number of 39.5 (IQR: 4.5–83), (all) men reported only a median of 13 (IQR: 0–51.5). This seems to be partly due to the much larger proportion of women reporting being married/cohabiting. In men 74.1, 15.5, and 10.4% of the sex acts were with cohabiting, non-cohabiting regular, and irregular, partners respectively. For women these percentages were 91.4, 4.5, and 4.1% respectively.

**Table 2:** Sexual behaviour as reported by a three months diary

	Sex	
	F (*N* = 148) %	M (*N* = 139) %
Any Sex with Cohabiting partner	77.70	64.70
Any Sex with Non-cohabiting Regular partner	22.30	44.60
Any Sex with Irregular Partners	12.20	33.80
Any Unprotected Sex with Cohabiting partner	77.00	61.90
Any Unprotected Sex with Non-cohabiting Regular Partners	13.50	35.30
Any Unprotected Sex with Irregular Partners	8.80	20.90
High Risk Sex	14.90	27.30
Total Number of Types of Partners		
0	11.50	12.20
1	68.90	50.40
2	15.50	19.40
3	4.10	18.00
Concurrency	11.50	23.00

#### Consistency and validity of the diary method

**Fig. 3:**
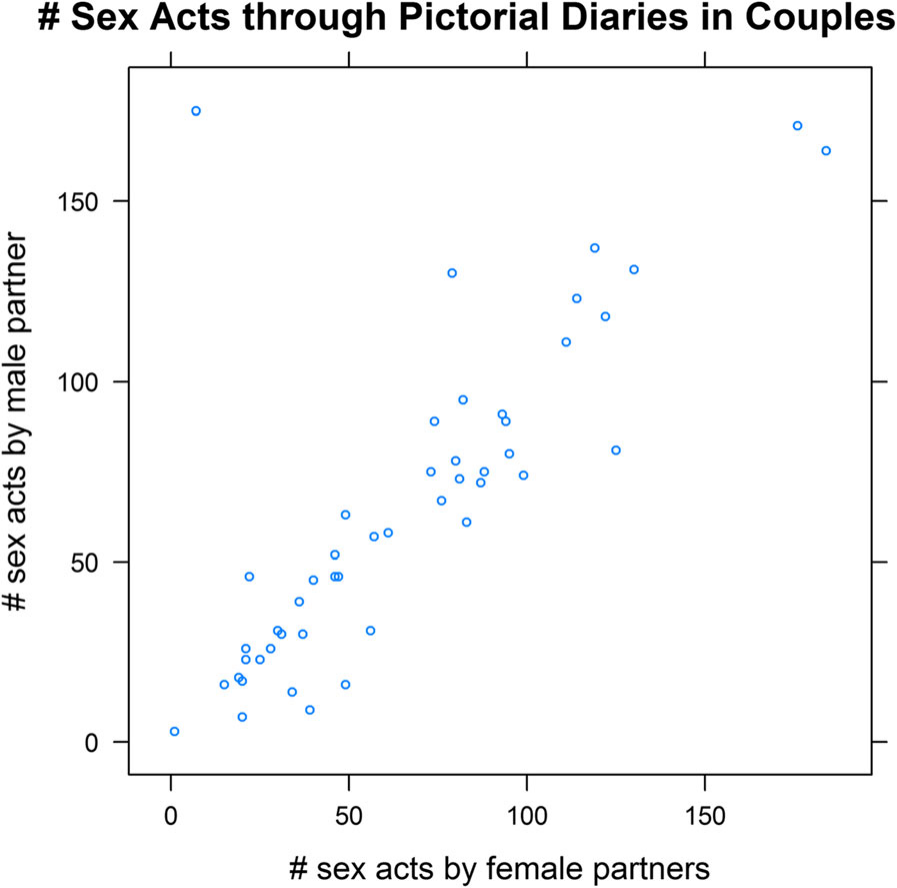
Association between numbers of sex acts over a 3-months period between two cohabiting partners reported through the pictorial diary

There was no discrepancy in the number of sex acts with the cohabiting partner in individuals in recruited couples. Numbers of sex acts reported by both partners within a couple appear to be in reasonable agreement as shown in the figure below (Fig. 3). Also, the number of condoms used did not differ significantly between male and female partners and, with a Spearman’s correlation coefficient of 0.72 was reasonably well correlated. The discrepancy in the number of sex acts discussed above thus seems to be largely attributable to more women than men being in cohabiting or marital relationships in our sample.

#### Type of sexual partners (cohabiting, regular, non-regular)

##### Cohabiting partners

More women (115/148) than men (90/139) reported (any) sex with a cohabiting partner. These numbers were actually larger than the numbers reporting to be married or cohabiting.

##### Regular non-cohabiting partners

More men (62/139) than women (33/148) reported sex with a non-cohabiting regular partner. Of these 32 and 17 respectively also reported sex with a cohabiting partner.

##### Irregular partners

More men (47/139) than women (18/148) reported any sex with an irregular partner. Of these 28 and 11 respectively also reported sex with a cohabiting partner.

The percentages of women reporting sex with 0, 1, 2, and 3 types (cohabiting, non-cohabiting regular, irregular) of partner were 11.5, 69.9, 15.5, and 4.1% respectively. For men these percentages were 12.2, 50.4, 19.4, 18.0%. Thus 19.6% of women and 37.4% of men reported more than one (type of) sexual partners by diary in the three-month study period.

#### High-risk behaviour

For participants with cohabiting partners any other partners would imply having multiple partners simultaneously. For participants without cohabiting partners, a regular, non-cohabiting partner however may be their only and monogamous partner. We therefore defined high risk behaviour differently for participants reporting sex with a cohabiting partner and those who did not. For the former we defined it as any unprotected (i.e. without condom use) sex with any regular or irregular partner; for the latter, not-cohabiting, group we defined it as unprotected sex with irregular partners only. Men reported higher levels of high-risk behaviour*,* viz 27.3% (38/139) than women 14.9% (22/148; *p* < 0.01). There was no clear association between HIV infection and high risk behaviour during the three-month diary period. 2/37 (5.4%) of the “high risk” men vs. 9/99 (9.1%) “low risk” men and 4/21 (19.0%) “high-risk” vs 12/123 (9.8%) “low-risk” women were HIV infected.

#### Concurrency

Having more than one regular partner was quite common in this cohort. Although we did not measure the total number of regular partners, we could count the number of individuals reporting both cohabiting and (at least one) additional regular partner. Overall 49 (17%) of individuals (23% of men, 11.5% of women) had concurrent relationships by this definition; slightly higher than observed elsewhere in Malawi (Likoma Island, a rural area in Northern Malawi) [25]. There was no apparent relationship between concurrency and HIV status, with 3/45 (vs 24/235) of the “concurrent participants” for whom HIV status was known being HIV positive.

#### Condom use by type of partner

Men reported a smaller fraction of condom-protected contacts with cohabiting partners (11.3%) than women (15.5%). Condoms were much more frequently used with non-cohabiting regular (F:75.7%; M:59.7%) and irregular partners (F:88.4%; M:74.1%).

#### Consistency of condom use

Of the 47 men reporting sex with irregular sex partners, 7 reported (by diary) that they never used condoms and 8 that they always used condoms. For women, of the 18 with irregular sex partners, 5 reported never use and 5 reported always use. For regular non-cohabiting partners condom use was also often inconsistent: of the 33 women reporting sex with regular non-cohabiting partners, 13 reported always using condom and 8 never using them. Of the 62 men, 12 reported always using and 9 never using condoms. It is unclear whether more than one regular non-cohabiting partner was involved and, if so, whether condom use differed among these partners.

### Agreement between self-reported partners in ACASI, FTFI and coital diaries

The number of *types* of sex partners (cohabiting, regular, irregular) reported (during the 3 months) by coital diary can be 0, 1, 2, or 3. This number should normally be a lower bound (as people may have multiple sex partners of the same type, which the diary does not distinguish) for the number of 3 months partners reported by either ACASI or FTFI. This was not true for a substantial number of participants. By ACASI 24 women and 33 men reported fewer partners than the presumed lower bound calculated from the diaries. For FTFI these numbers were 24 and 40 respectively. It may be that participants had problems remembering when specific events (relationships) took place, or simply forgot about them, or that partners changed “status” during the 3 months period.

## Discussion

Accurate measurement of sexual behaviour, notably unprotected sexual intercourse with multiple and non-steady partners is critical in HIV prevention research in order to enable attribution of changes in incidence within specific contexts. Sexual behaviours are challenging to measure and data triangulation is an important element in assessing both data validity and quality [26]. Our data triangulation study, comparing two retrospective tools to a prospective pictorial diary, showed only moderate agreement between three different self-reported measures of sexual behaviour. While number of partners reported in our two retrospective tools ACASI and FTFI did not differ systematically, and were positively correlated, their correlation was far from perfect. Even seemingly straightforward questions about whether one has ever had sex and about marital status did not always yield identical answers, perhaps indicating some ambiguity or misinterpretation in what constituted “sex” or what exactly is a spouse/cohabiting partner**.** Agreement between data reported through the two retrospective measures of self-reported sexual behaviour and the three-month diary method was also far from perfect. It is perhaps most striking that the percentages of participants reporting more than one sexual partner in the past 3 months was substantially higher by diary than by the two retrospective end-of-period self-reporting methods. This has been found in other studies in SSA as well [16, 17, 19] and may be due to recall or social desirability bias.

Our findings conflict with those from a recent systematic review that reported that studies comparing diary with retrospective survey data demonstrated “evidence of over-reporting on retrospective tools, except for the least frequent behaviours” [27] such as unprotected insertive anal sex for Men who have Sex with Men (MSM). However, our findings agree with those from other African countries, including those from nearby Zimbabwe that both retrospective instruments, even for short term recollection, are equally poor and may significantly underestimate true sexual activity [28, 29]. Other reasons for the discrepancies between our diary and retrospective tools are hard to identify with certainty.

Context matters when recalling sexual interactions. The commonly used three-month recollection period of the retrospective instruments may be too long and thus be a factor contributing to these discrepancies. It may also be that our participants, from an African culture where time may have a different role than in industrialized countries, had more difficulty establishing whether certain events or relationships took place within the past 3 months or not (http://www.exactlywhatistime.com/time-in-different-cultures) . It seems less likely that social desirability bias also played an important role as the numbers of partners reported by the “anonymous” ACASI and FTFI did not differ systematically. Since the diary method does not require participants to reveal aggregate numbers of sex partners on the basis of their memory but asks them to report on a day-by-day basis, the diary method may well be more precise than either the ACASI or the FTFI method. There tended to be a high level of agreement between diaries of participating couples, which seems to add some validity to the diary method. Some couples, however, might have harmonized their responses, although the variation that occurred suggests otherwise. Perhaps, in some African contexts, (pictorial) diaries may thus, for the time being, be the preferred method for eliciting sexual behaviour.

Further validation of the diary method in this setting is necessary. Ideally, diaries should be compared to the same biomarkers that have demonstrated the limited value of self-reported retrospective data even over very short recollection periods [30]. Our findings, especially the higher percentages of participants who reported > 1 partner in the past 3 months with the diary method, would seem to justify such further validation of this method. Exploration as to whether more detailed information about sex partners could be obtained using this method would also be worthwhile. Understanding aspects, such as whether a sexual partner is a new partner, or ages of partners, could be valuable information for understanding the unfolding HIV epidemic.

A limitation of our sample is that it may not be truly representative of the underlying population since we re-contacted only individuals accepting HIVST and who were recruited within the context of the larger study. Given the higher proportion of married women than men, some self-selection may have taken place, with perhaps unmarried women and/or married men being less willing to participate. It is hard to tell how much this may have affected or biased our estimates.

What emerges, despite these limitations, is a relatively young population with high levels of multiple-partner sex and sexual risk taking. The percentages of multiple partner sex (F:19.6%, M:37.4% by diary) appear not to be substantially higher than those among sexually active (i.e. having had sex in the past 3 months) adolescents and young adults in the USA, where 15% of women and 35% men reported multiple sex partners in the past 3 months [31]. It may be that this level of risk taking can be sufficient to establish large connected sexual networks. The Likoma Island (rural Malawi) study demonstrated that “sexual networks emerged through decentralized chains of sexual relationships in which individuals had at most three to four sexual partners over a 3-year period, rather than through contacts with high-risk groups such as commercial sex workers” [32]. Condoms appear to be acceptable in this population, and as expected and appropriate, were most frequently used with non-cohabiting partners. However, inconsistent use may limit their potential. As condom use was often not consistent its impact on HIV transmission may be limited. Identifying methods for improving consistent use, should be a research priority.[33]

## Conclusion

This study has shown that, without biological markers, it is challenging to define an optimal or gold standard method for collection of reliable data on sensitive behaviours such as sexual risk behaviour. Context and gender-driven influences on acceptability and reporting of sexual behaviour impacts on all reporting methods, including daily recording of sexual acts and partners through pictorial diaries. Despite these challenges, we suggest that daily recording of sensitive behaviours may be most accurate and that this method should be considered as an option for measuring sensitive behaviours, especially linked to HIV prevention technologies, in both general and vulnerable populations.

## Additional file

Additional file 1: Data tools. (ZIP 2282 kb)

## Abbreviations

ACASI: Audio computer assisted self interviews
ART: Antiretroviral therapy
FTFI: Face to face interview
HIV: Human immunodeficiency virus
HIVST: HIV self-testing
IQR: Inter-quartile range
MSM: Men who have sex with men
PrEP: Pre exposure prophlyaxis
UTT: Universal test and treat

## Footnotes

1 Full diary completion for each participant entailed 6 diaries, each of which covered a 2 week period for the 12 weeks (3 months) of the study period
