# Supplementary material for: Measuring sexual behaviour in Malawi: a triangulation of three data collection instruments
Source: BMC Public Health. 2018 Jun 28;18:807. doi: 10.1186/s12889-018-5717-x (PMC6022416; doi:10.1186/s12889-018-5717-x)
Supplement: Supplementary file 1 — Data tools. (ZIP 2282 kb) [file 12889_2018_5717_MOESM1_ESM.zip › FullElectronicDiary_FinalR2.pdf]

LDS /☐☐☐/ ☐

Participant ID   Complete if couple
Insert M/F

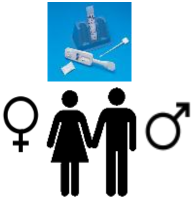

ST Impacts: social impacts of HIV self-testing

Pictorial diary study

Start date: ☐☐/☐☐/☐☐☐☐

End date: ☐☐/☐☐/☐☐☐☐

Fieldworker ID: ☐☐

Diary completion instruction sheet

This is a reminder to help you fill in this diary. This diary lasts for two weeks and contains one laminated sheet. You will receive a new diary every two weeks for 12 weeks.

1. Please complete the daily sheet every day.
2. Complete the weekly sheet during the week or at the end of each week.
3. You will be visited regularly throughout the time you are completing diaries so please feel free to ask any questions when we meet with you.
4. For daily diaries, column A should be completed in all cases. Please circle the tick if an event occurred and complete details along the line in column B. Circle the cross if no event occurred and do not complete along the line in column B.
5. For weekly diaries, column A should be completed in all cases. Please circle the tick if an event occurred and complete details along the line in column B. Circle the cross if no event occurred and do not complete along the line in column B.
6. Keep completed diaries in a secure location and return to the fieldworker when she/he visits.

We would like to thank you for completing this diary. If you have any questions about this diary please contact the study team on telephone number 0995351611/0999981937 or the study Principal investigator on telephone number 0999016850.

Date: ☐☐/☐☐/☐☐☐☐

LDS /☐☐☐/ ☐

Participant ID Complete if couple
Insert M/F

A	B
✓<br>✗	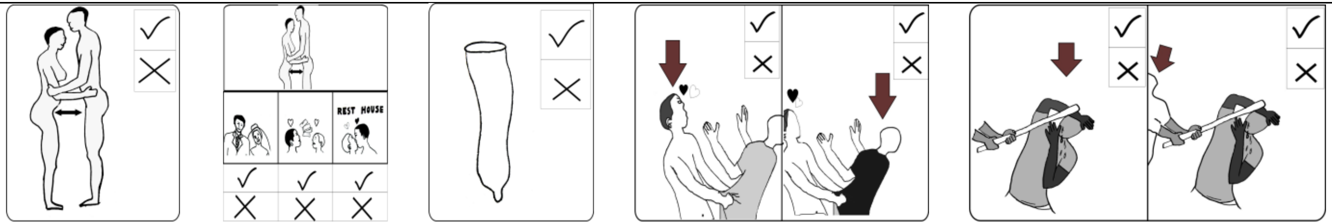
✓<br>✗	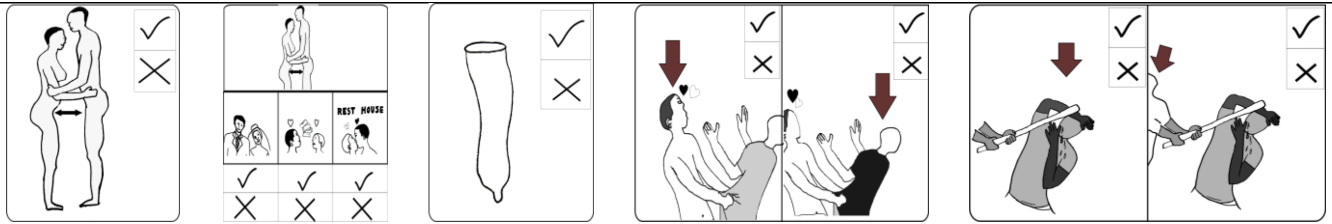
✓<br>✗	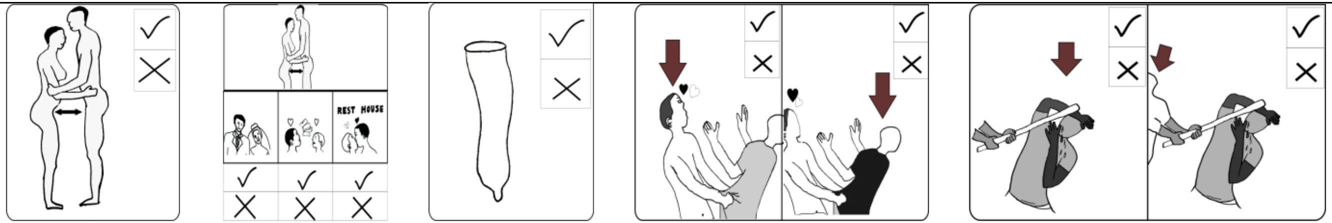

Date: ☐☐/☐☐/☐☐☐☐

LDS /☐☐☐/ ☐

Participant ID Complete if couple
Insert M/F

A	B
✓<br>✗	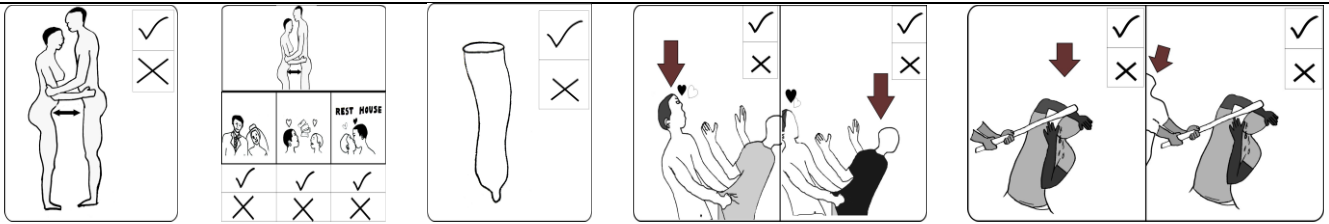
✓<br>✗	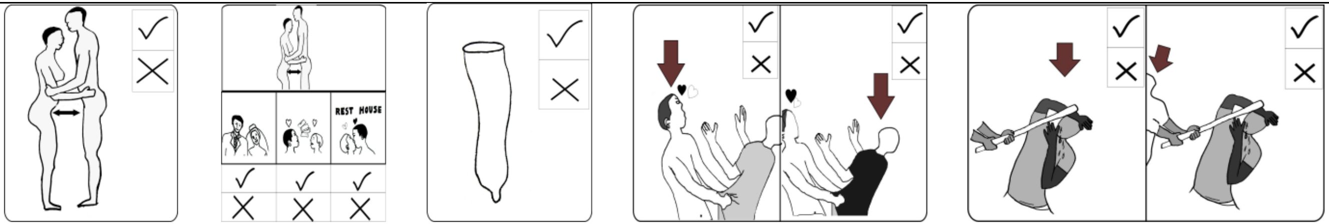
✓<br>✗	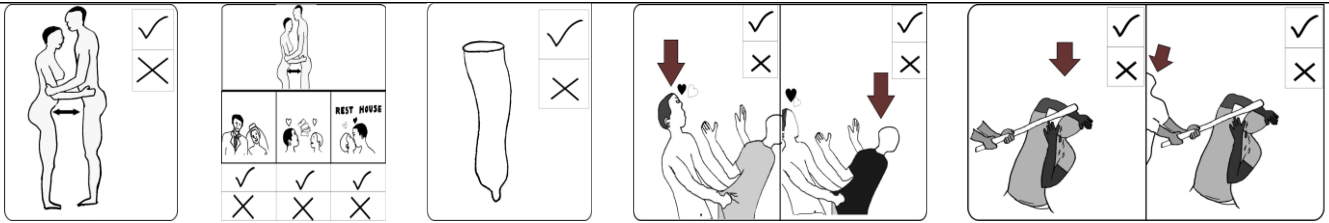

Date: □□/□□/□□□□

LDS /□□□/ □

Participant ID Complete if couple Insert M/F

A	B
✓<br>✗	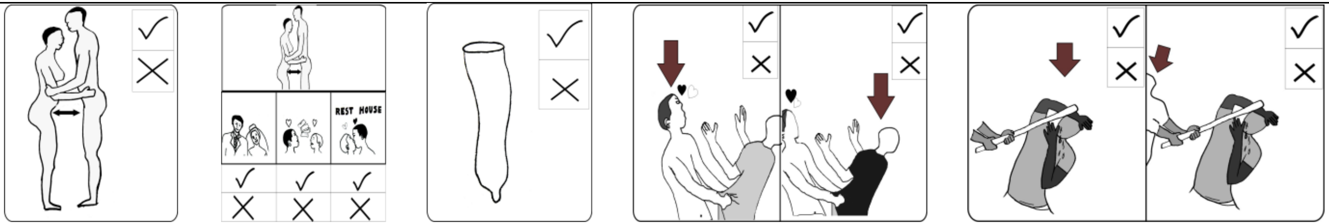
✓<br>✗	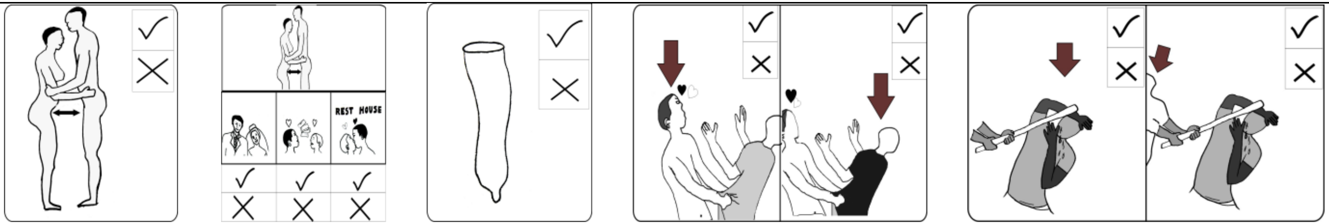
✓<br>✗	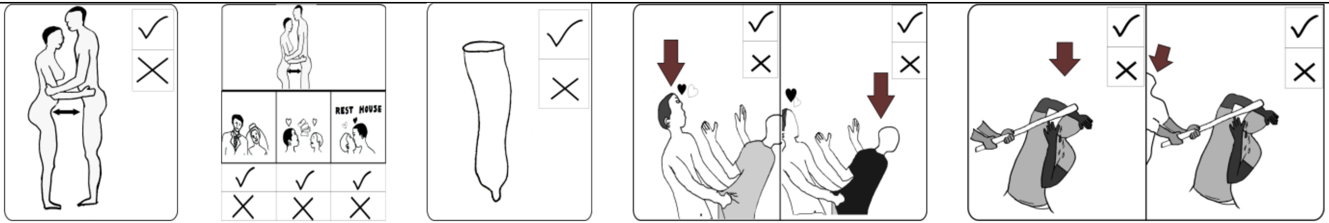

Date: ☐☐/☐☐/☐☐☐☐

LDS /☐☐☐/ ☐

Participant ID   Complete if couple Insert M/F

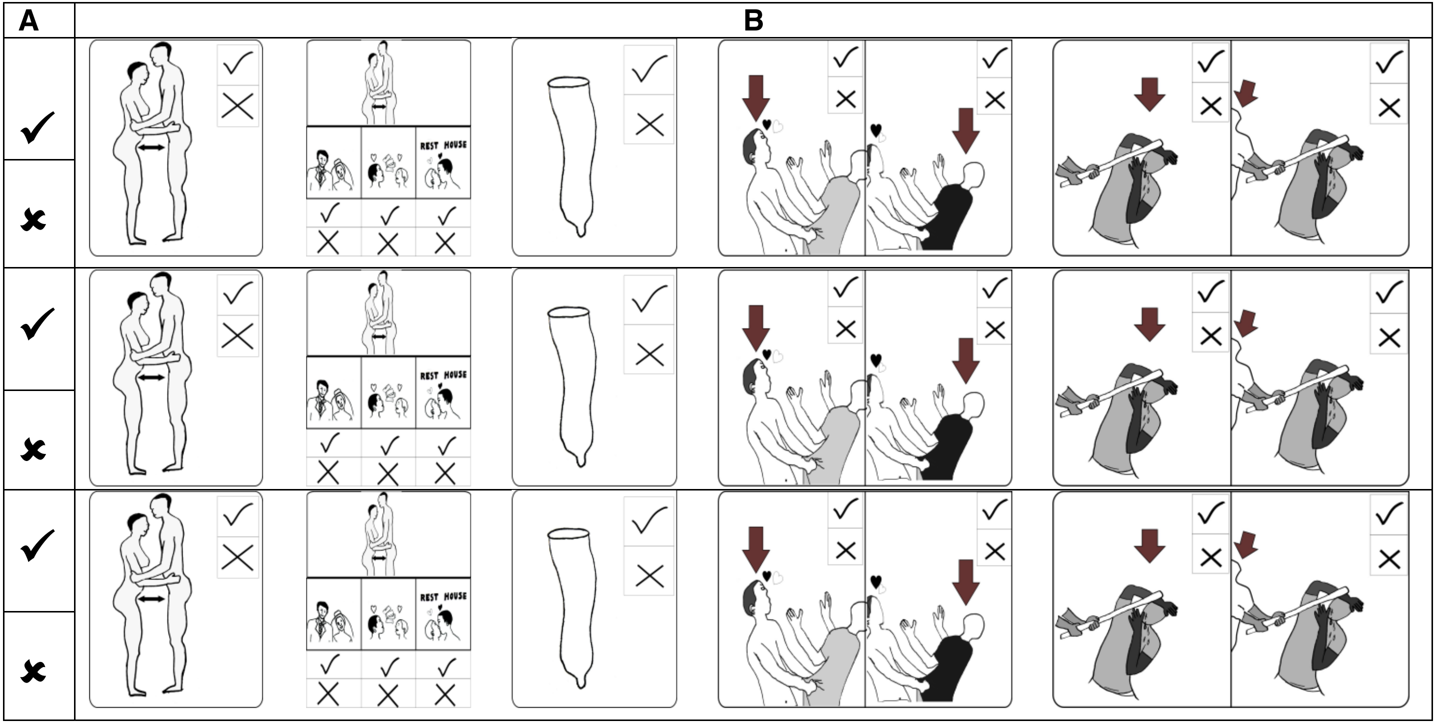

Date: □□/□□/□□□□

LDS /□□□/ □

Participant ID   Complete if couple
Insert M/F

A	B
✓<br>✗	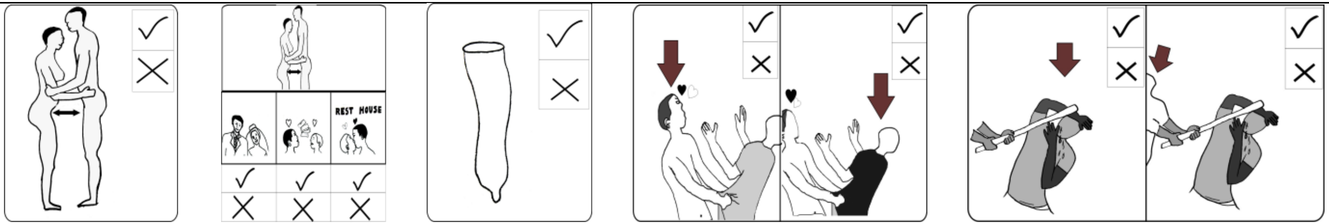
✓<br>✗	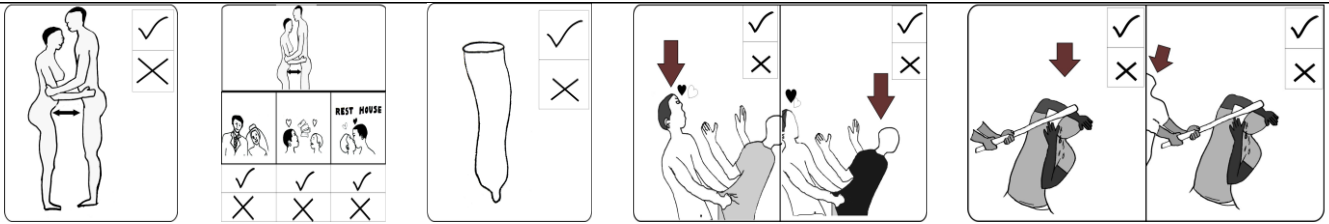
✓<br>✗	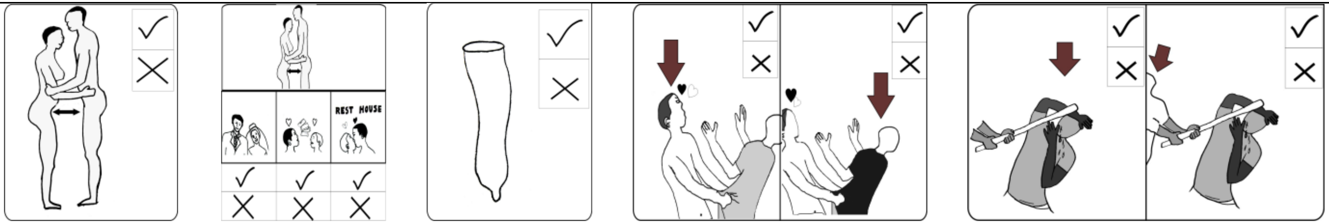

Date: ☐☐/☐☐/☐☐☐☐

LDS /☐☐☐/ ☐

Participant ID   Complete if couple
Insert M/F

A	B
✓	
✗	
✓	
✗	
✓	
✗	

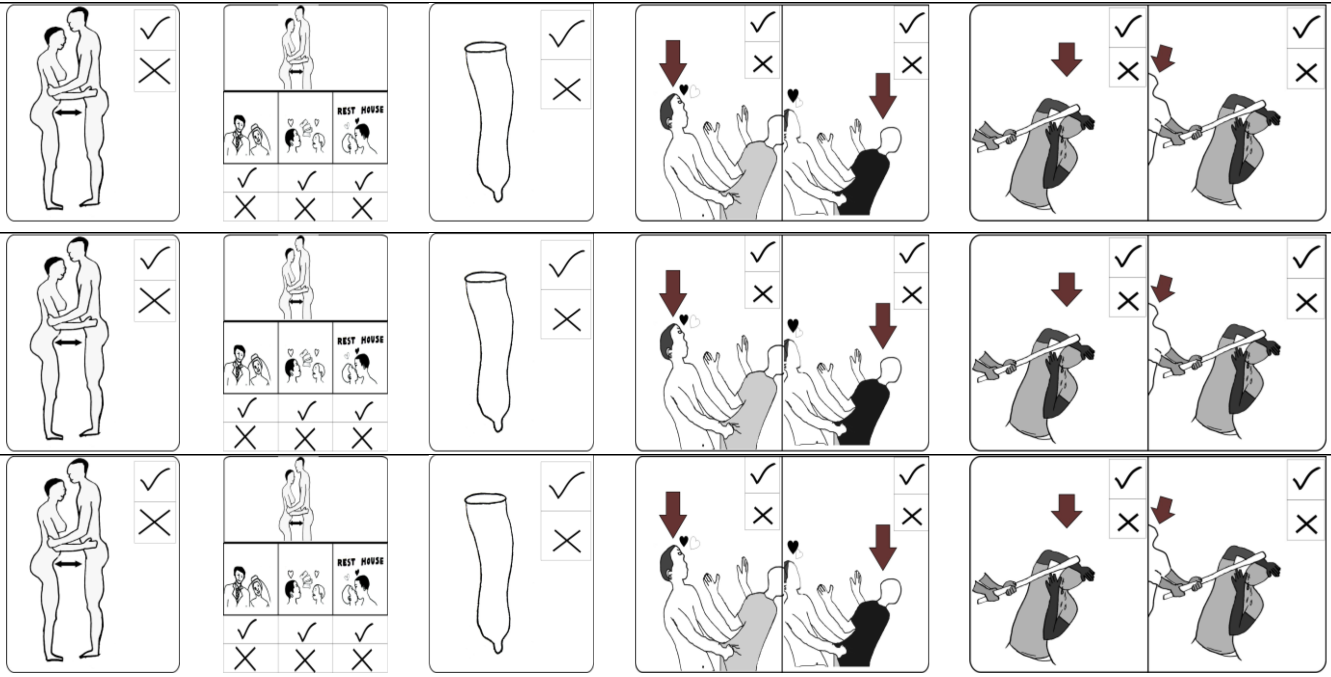

Date: □□/□□/□□□□

LDS /□□□/ □

Participant ID   Complete if couple
Insert M/F

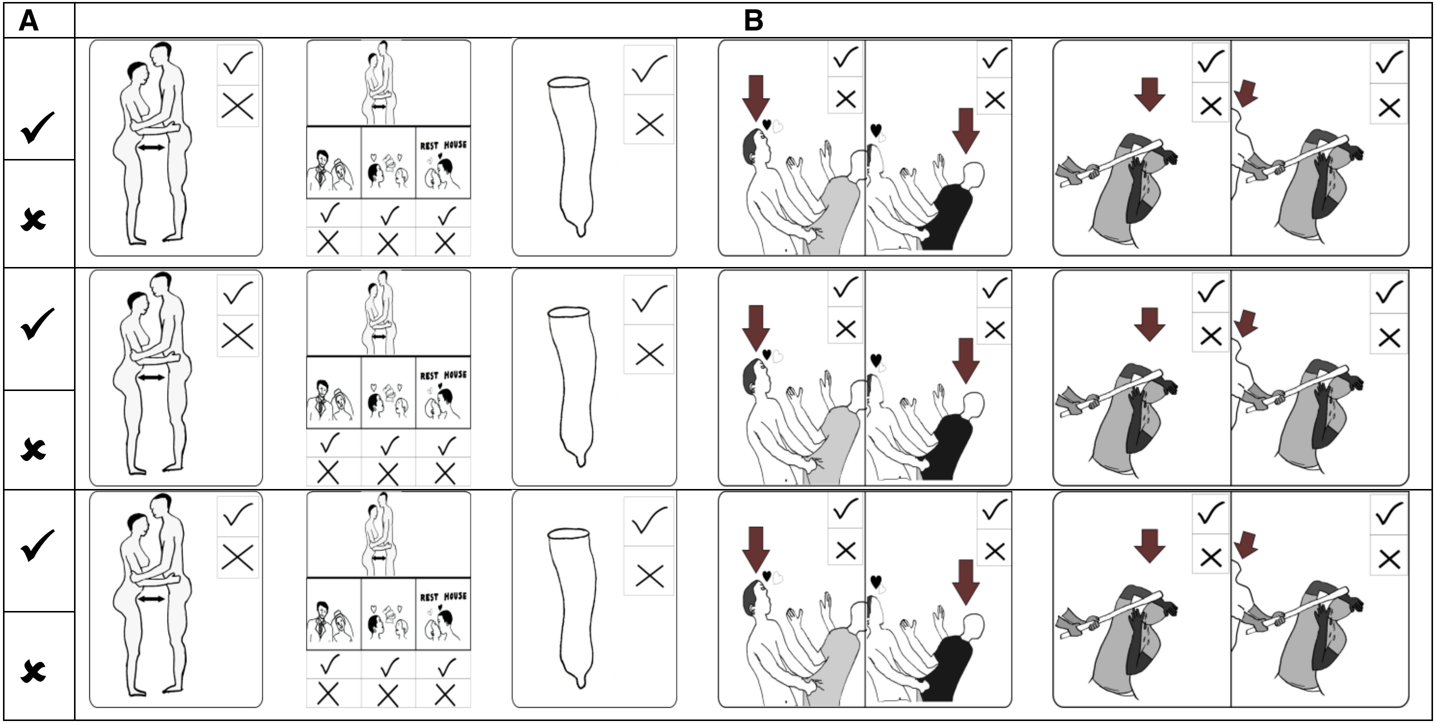

Date: □□/□□/□□□□

LDS /□□□/ □

Participant ID   Complete if couple
Insert M/F

A	B
✓	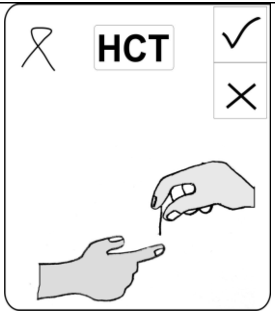 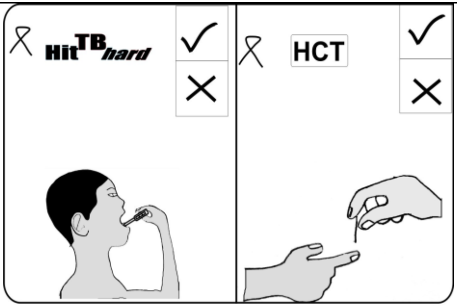 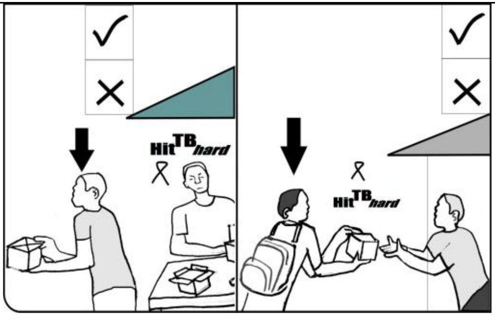
✗	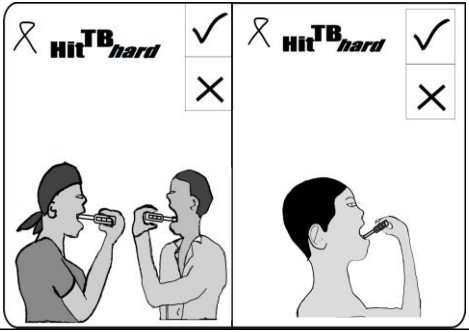 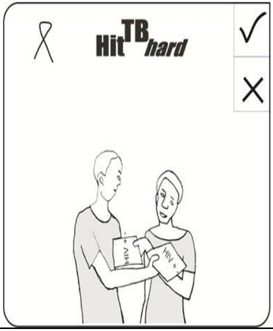 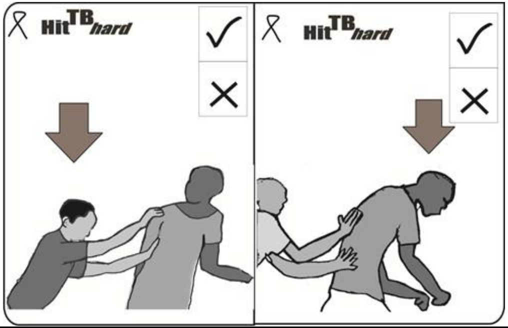

Date: □□/□□/□□□□

LDS /□□□/ □

Participant ID   Complete if couple Insert M/F

A	B		
✓	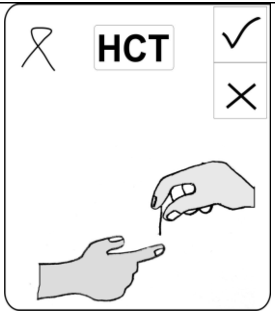	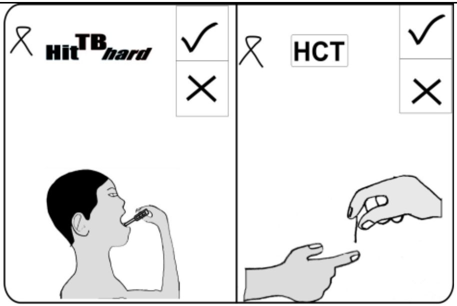	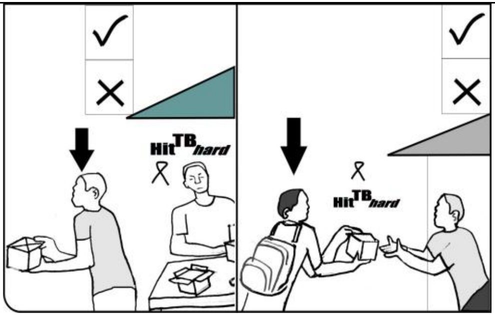
✗	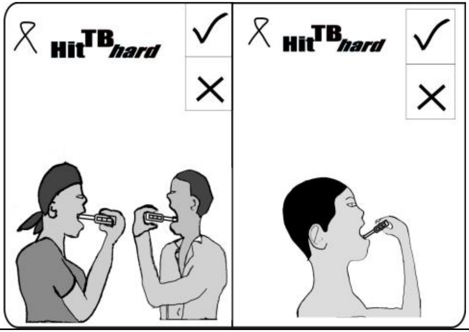	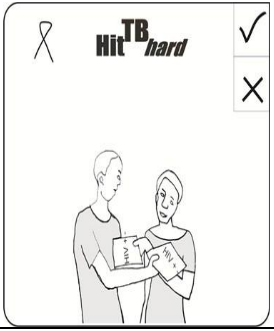	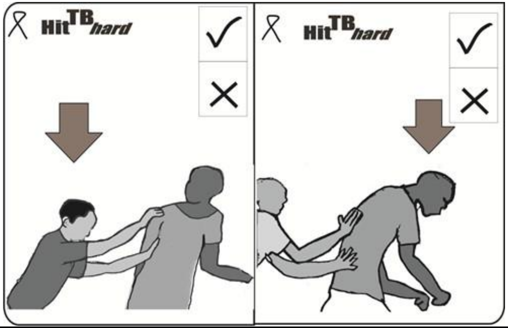

Date: □□/□□/□□□□

LDS /□□□/ □

Participant ID Complete if couple Insert M/F

A	B
✓	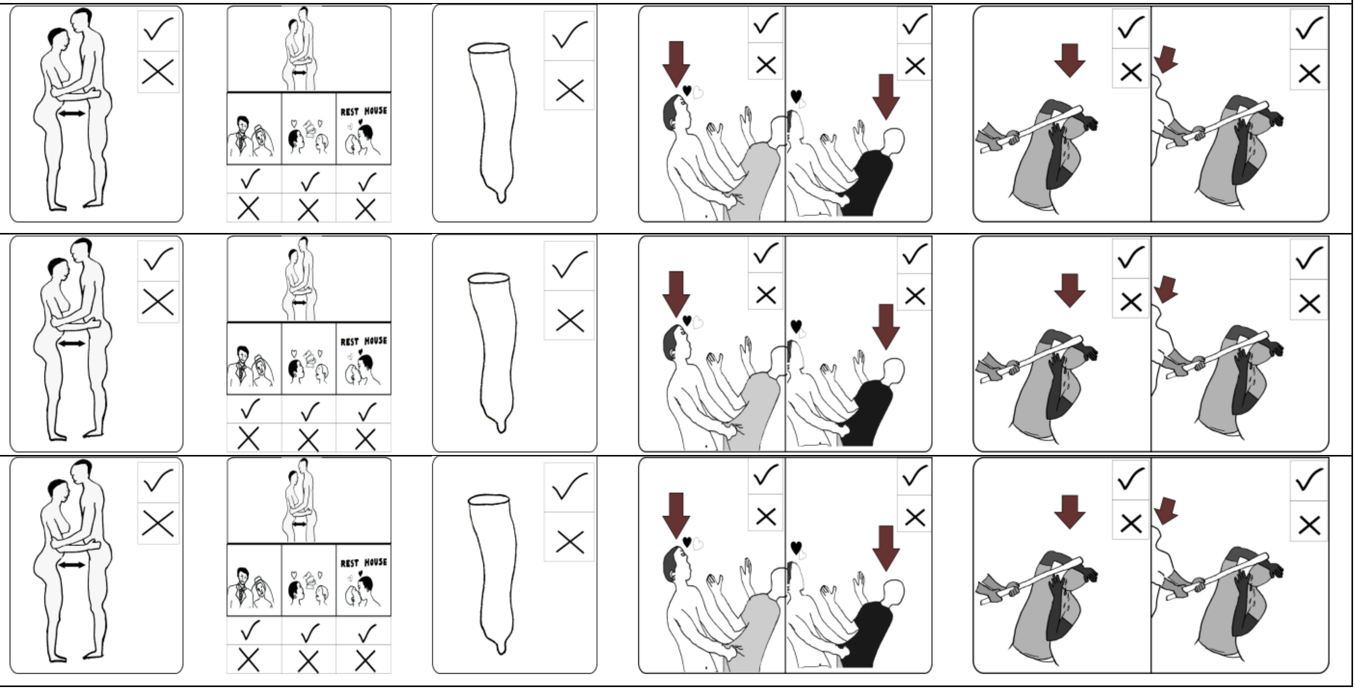
✗	
✓	
✗	
✓	
✗	

Date: ☐☐/☐☐/☐☐☐☐

LDS /☐☐☐/ ☐

Participant ID   Complete if couple
Insert M/F

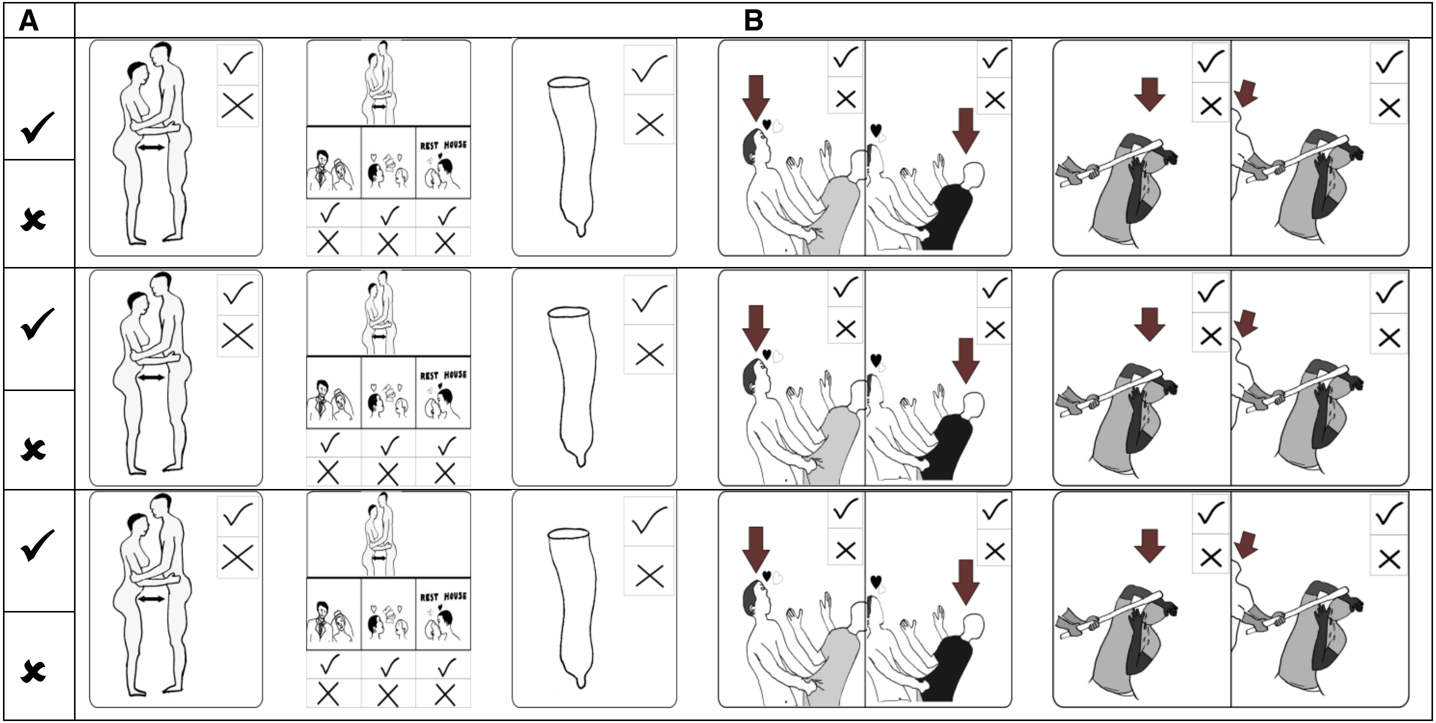

Date: ☐☐/☐☐/☐☐☐☐

LDS /☐☐☐/ ☐

Participant ID Complete if couple Insert M/F

A	B
✓<br>✗	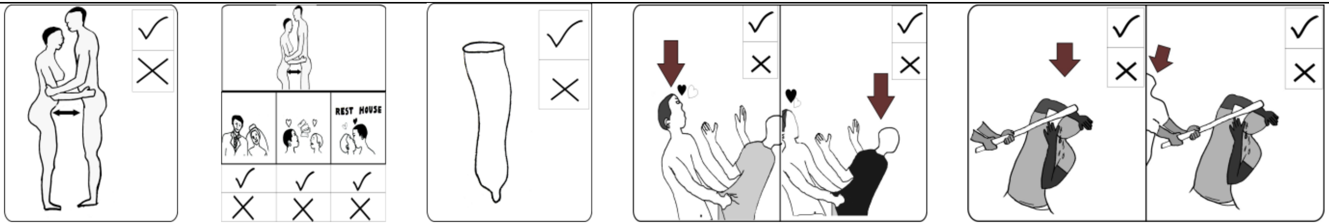
✓<br>✗	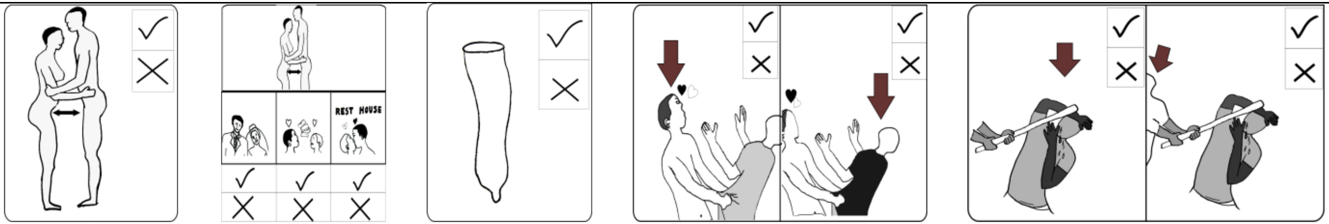
✓<br>✗	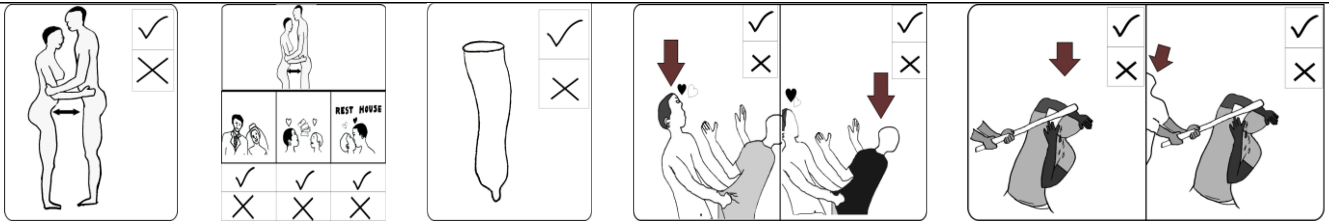

Date: ☐☐/☐☐/☐☐☐☐

LDS /☐☐☐/ ☐

Participant ID Complete if couple
Insert M/F

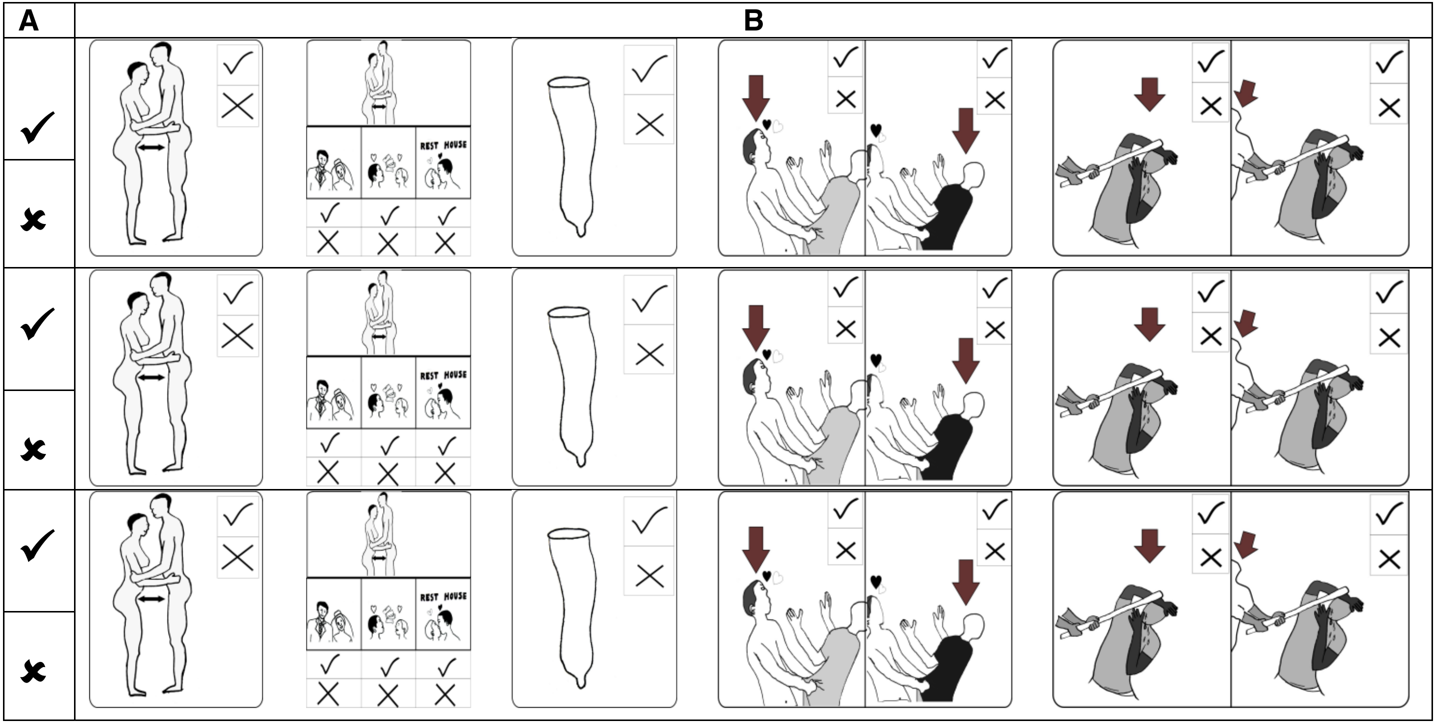

Date: ☐☐/☐☐/☐☐☐☐

LDS /☐☐☐/ ☐

Participant ID   Complete if couple
Insert M/F

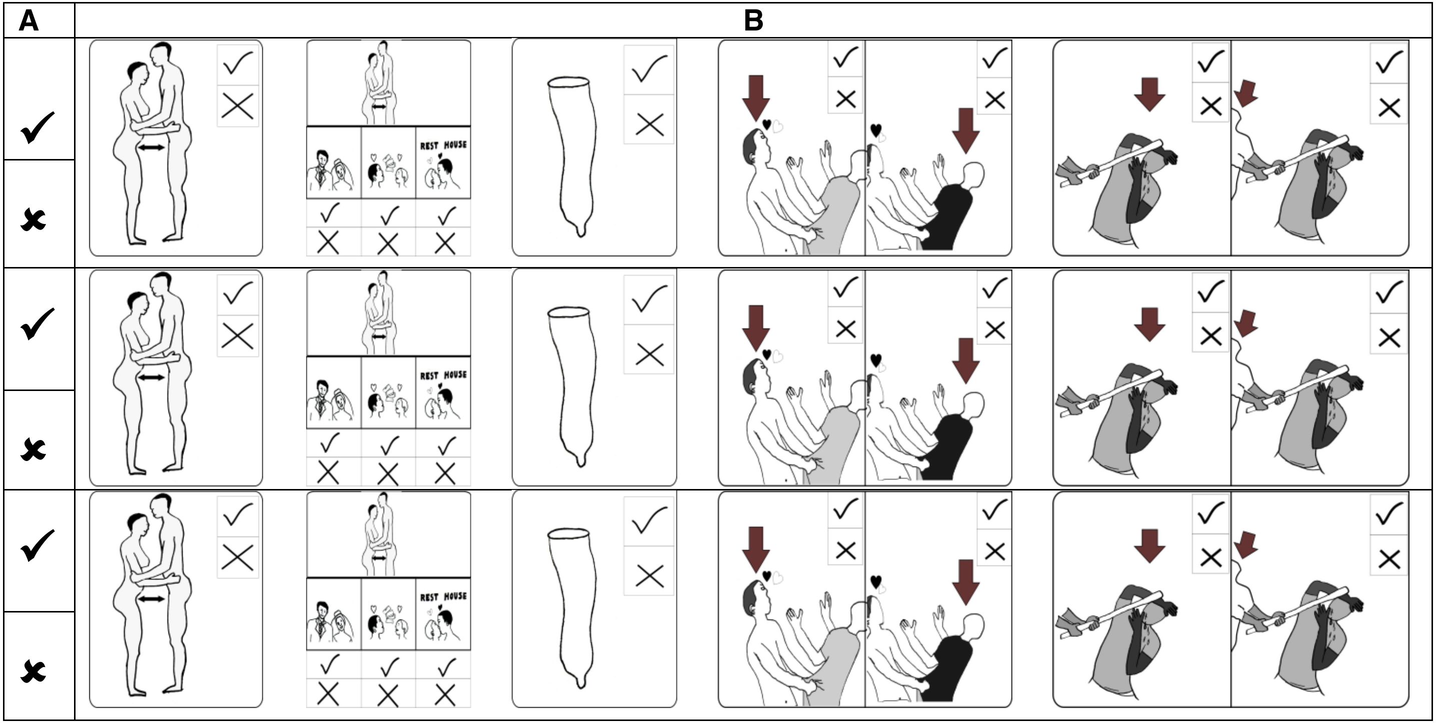

Date: □□/□□/□□□□

LDS /□□□/ □

Participant ID Complete if couple Insert M/F

A	B
✓<br>✗	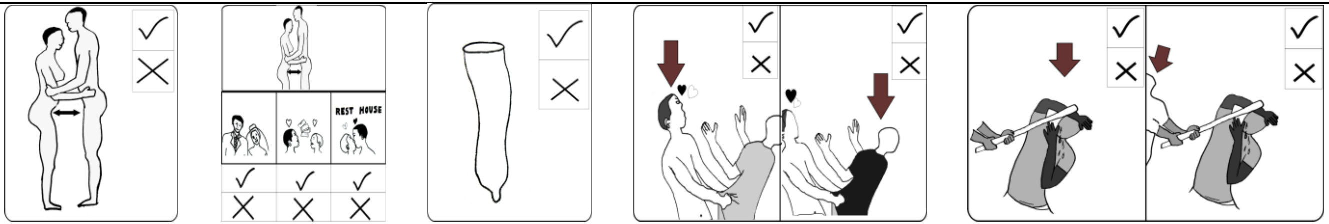
✓<br>✗	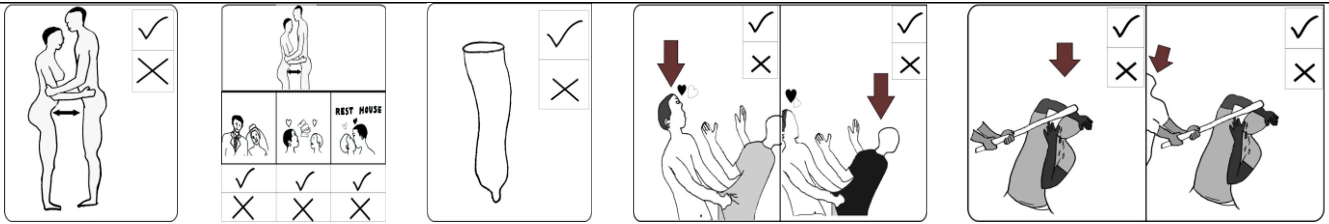
✓<br>✗	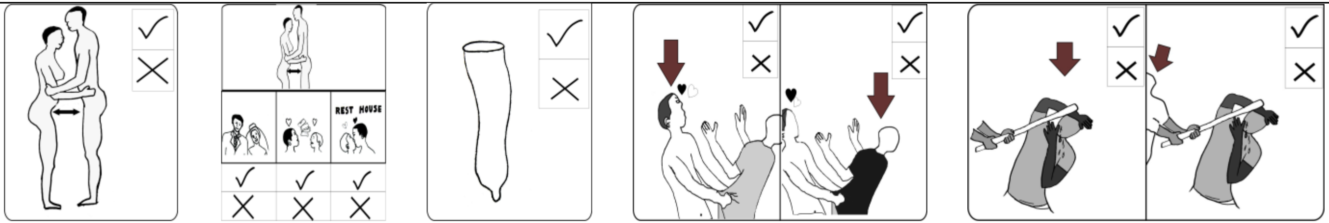

Date: □□/□□/□□□□

LDS /□□□/ □

Participant ID Complete if couple Insert M/F

A	B
✓ / ✗	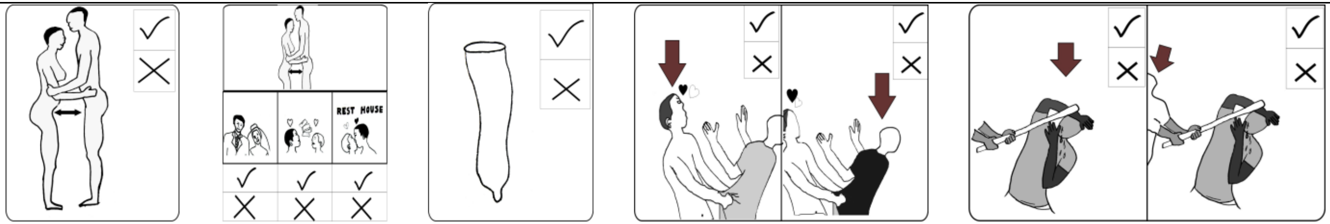
✓ / ✗	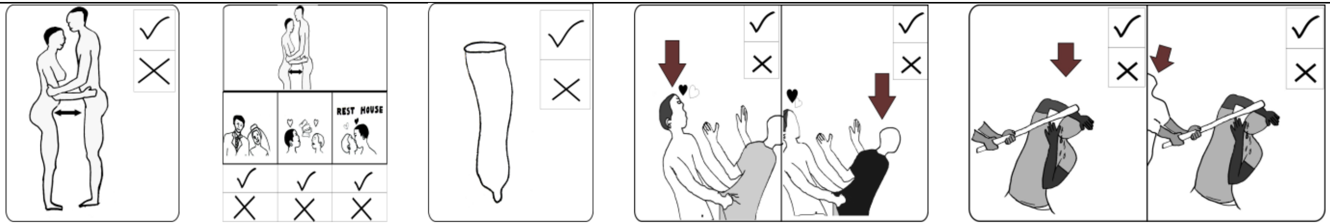
✓ / ✗	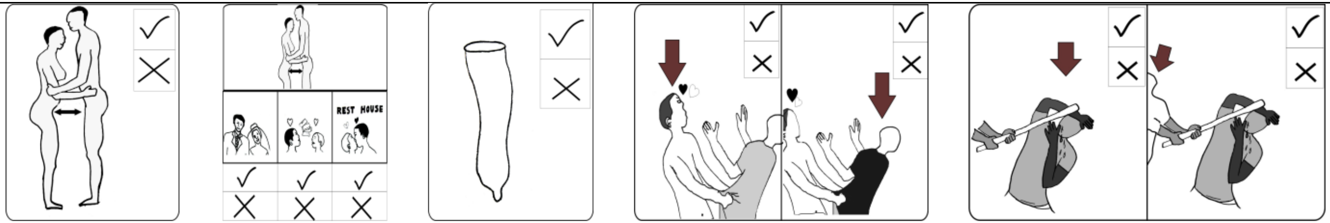

Date: ☐☐/☐☐/☐☐☐☐

LDS /☐☐☐/ ☐

Participant ID Complete if couple Insert M/F

A	B		
✓	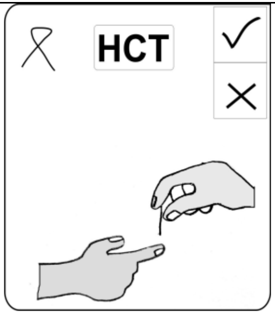	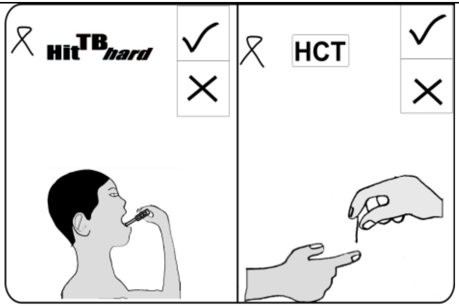	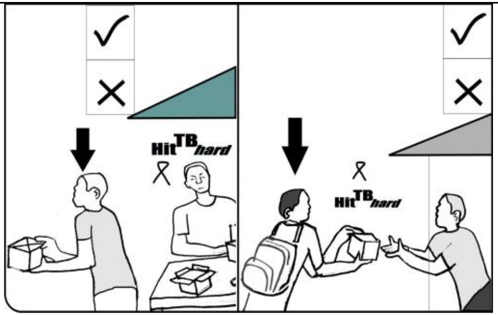
✗	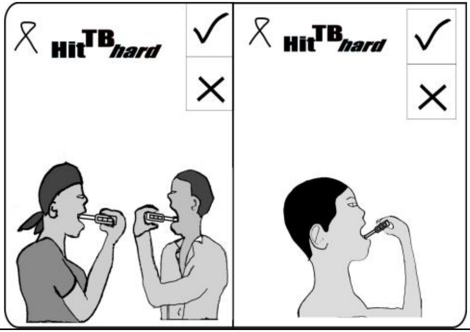	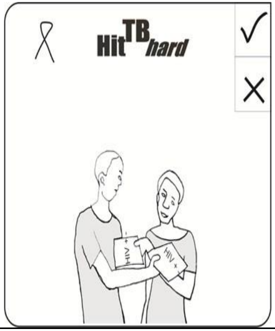	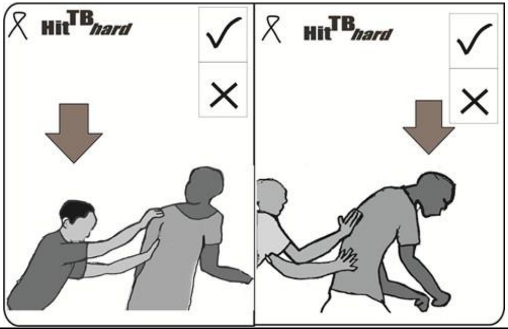

Date: ☐☐/☐☐/☐☐☐☐

LDS /☐☐☐/ ☐

Participant ID Complete if couple Insert M/F

A	B
✓	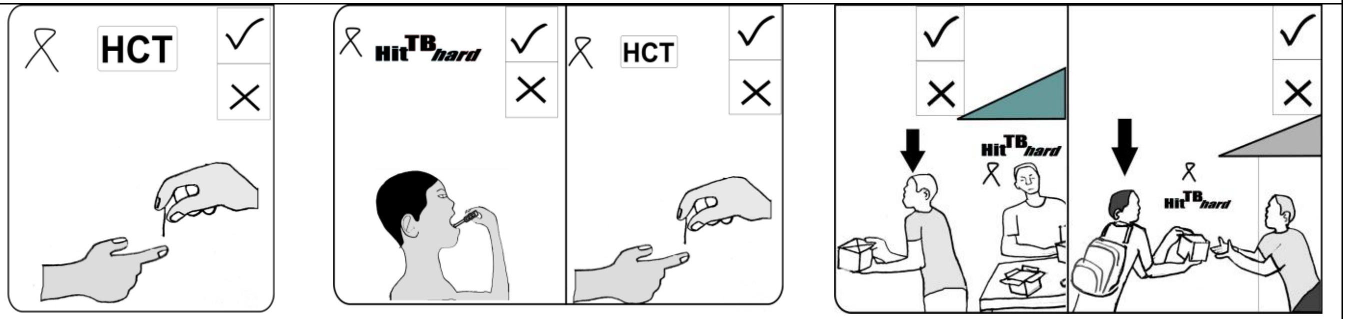
✗	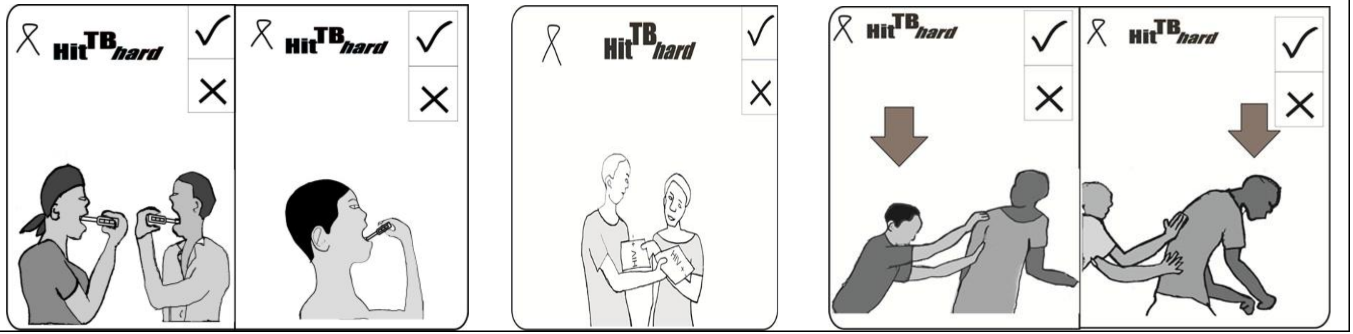
