# Supplementary material for: Measuring sexual behaviour in Malawi: a triangulation of three data collection instruments
Source: BMC Public Health. 2018 Jun 28;18:807. doi: 10.1186/s12889-018-5717-x (PMC6022416; doi:10.1186/s12889-018-5717-x)
Supplement: Supplementary file 1 — Data tools. (ZIP 2282 kb) [file 12889_2018_5717_MOESM1_ESM.zip › LDS 01_ACASI_FinalR2.docx]

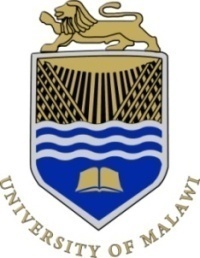

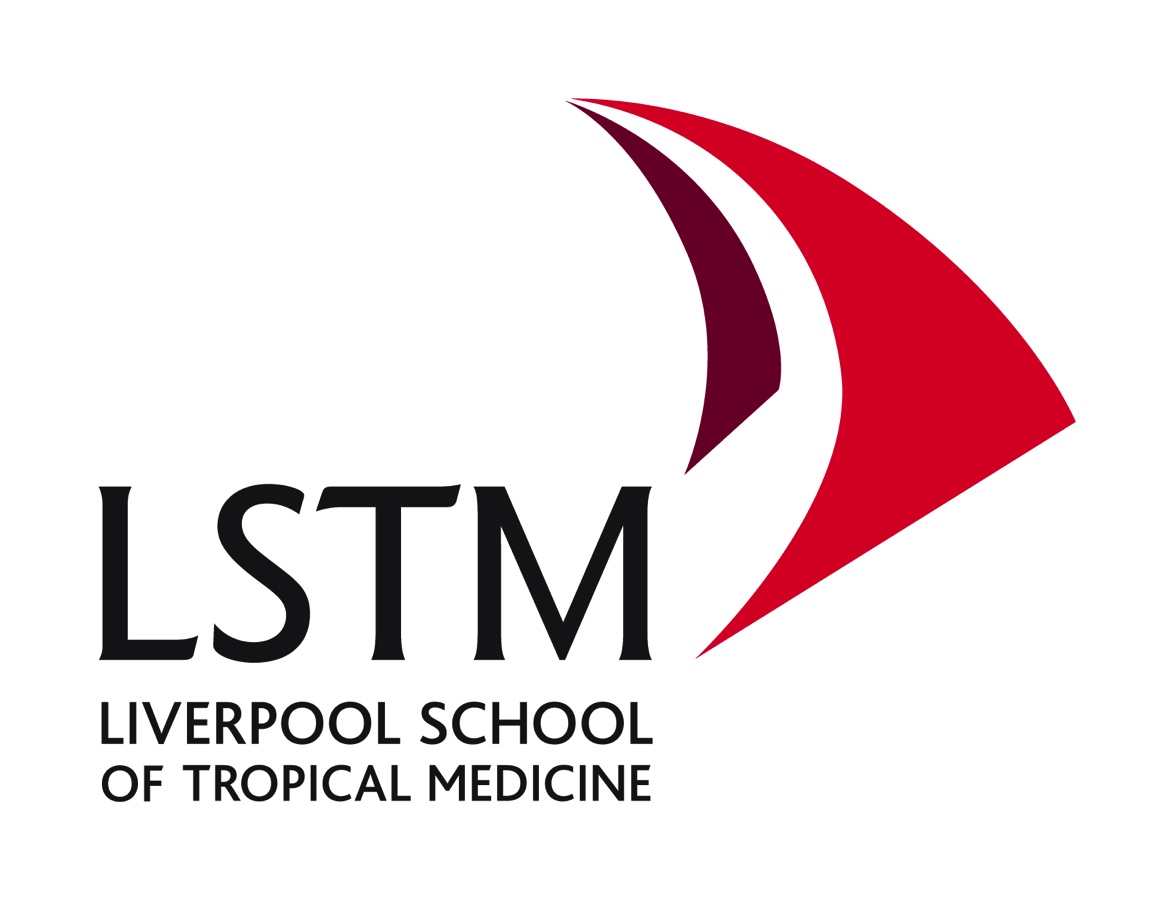

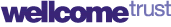

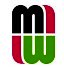


*Write ID number here*

DS /


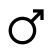

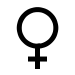

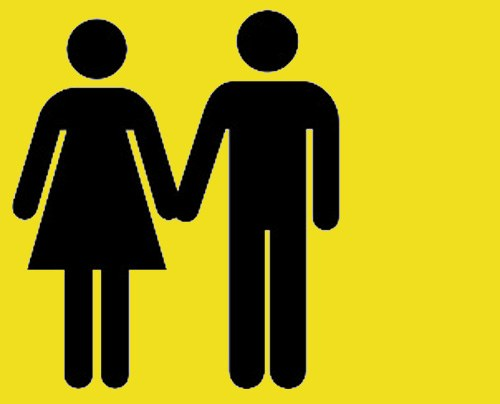


**LDS02: ST Impacts ACASI Variables**

| **Details of Interview** ***To be completed by Researcher*** | | | | | | | | | | | | |
| --- | --- | --- | --- | --- | --- | --- | --- | --- | --- | --- | --- | --- |
| Date of Interview | | | Researcher ID | | | | | | | | | |
| **Section A: Demographic Details *To be completed for all participants***  *‘First I am going to ask you some questions about yourself and your life’ (Section A and B)* | | | | | | | | | | | | |
| A05 | How would you describe your current marriage status? | NEVER MARRIED ………………………………………1  MARRIED OR LIVING WITH PARTNER ……….2  REMARRIED AFTER DIVORCE/DEATH ……….3  DIVORCED/SEPARATED …………………………….4  WIDOWED ……………………………………………….5 | | | | | | 1  2  3  4  5 | | | | |
| A06 | How many living children do you have? | NUMBER ............................... | | | | | |  | | | | |
| A07 | Of all your living children how many are currently dependent on you? | NUMBER ............................... | | | | | |  | | | | |
| **Section B: Individual behaviour** | | | | | | | | | | | | |
| B01 | How many times have you been married in your lifetime including (if relevant) your current marriage? | YEARS ……………………………… | | | | | |  | | | | |
| B02 | How many sexual partners have you had in your lifetime? | NUMBER ............................... | | | | | |  | | | | |
| B03 | In the past three months how many sexual partners have you had? | NUMBER ............................... | | | | | |  | | | | |
| B04 | Of your partners in the last 3 months how many were regular partners *(a regular partner is someone who you have sex with often and who provides support to meet your daily needs – they may or may not live with you*) | NUMBER ............................... | | | | | |  | | | | |
| B05 | How many days is it since you last had sex? | NUMBER ................................ | | | | | |  | | | | |
| B06 | With whom did you have sex most recently? | HUSBAND/ PARTNER RESIDENT…………1  REGULAR PARTNER NON-RESIDENT.....2  IRREGULAR PARTNER…………………………3  OTHER (specify)……………………………....99  REFUSED TO ANSWER……………………..77 | | | | | | 1  2  3  99  77 | | | | |
| B07 | Did you use a condom last time you had sex? | YES ……………………………………………………1  NO ……………………………………………………2  REFUSED TO ANSWER …………………….77 | | | | | | 1  2  77 | | | | |
| B08 | In the past 3 months how regularly have you used a condom when having sex? | EVERY TIME …………………………………….1  SOMETIMES ……………………………………2  NEVER …………………………………………….3 | | | | | | 1  2  3 | | | | |
| B09 | Do you sometimes drink alcohol | YES ……………………………………………………1  NO ……………………………………………………2  REFUSED TO ANSWER …………………….77 | | | | | | 1  2  77 | | | | |
| B10 | In the past 3 months how often have you had drinks containing alcohol? | EVERY DAY.....................................1  AT LEAST ONCE A WEEK……………...2  AT LEAST ONCE A MONTH.............3  AT LEAST ONCE IN 3 MONTHS …….4  NEVER............................................5 | | | | | | 1  2  3  4  5 | | | | |
| B12 | In the last 3 months how often did you drink alcohol before having sex? | NEVER ..................................1  ONCE ....................................2  2-4 TIMES .............................3  MORE THAN 4 TIMES ...........4  REFUSED TO ANSWER ........77  DON’T KNOW .....................88 | | | | | | 1  2  3  4  77  88 | | | | |
| B15 | Have you previously self-tested through HitTB? | YES ......................................1  NO.........................................2 | | | | | | 1  2 | | | | |
| B16 | Other than self-testing with HitTB, have you previously tested for HIV in your lifetime? | YES ……………………………………………………1  NO ……………………………………………………2  REFUSED TO ANSWER …………………….77 | | | | | | 1  2  77 | | | | |
| B17 | Other than self-testing with HitTB, how many times have you tested for HIV in your lifetime? | NUMBER …………………………………….. | | | | | |  | | | | |
| B18 | Other than self-testing with HitTB, have you previously tested for HIV with a partner in your lifetime *(couples testing)?* | YES ……………………………………………………1  NO ……………………………………………………2  REFUSED TO ANSWER …………………….77 | | | | | | 1  2  77 | | | | |
| B20 | Have you ever been persuaded to test for HIV? | YES ……………………………………………………1  NO ……………………………………………………2 | | | | | | 1  2 | | | | |
| B21 | If yes, who persuaded you to test for HIV? | PARTNER ………………………………………….1  OTHER FAMILY MEMBER ………………….2  FRIEND/NEIGHBOUR ………………………..3  HEALTH WORKER …………………………….4  OTHER *(Please specify) ………………………*.99 | | | | | | 1  2  3  4  99 | | | | |
| B22 | Have you ever been forced to test for HIV? | YES ……………………………………………………1  NO ……………………………………………………2 | | | | | | 1  2 | | | | |
| B23 | If yes, who forced you to test for HIV? | PARTNER ………………………………………….1  OTHER FAMILY MEMBER ………………….2  FRIEND/NEIGHBOUR ………………………..3  HEALTH WORKER …………………………….4  OTHER *(Please specify) ……………………*.99 | | | | | | 1  2  3  4  99 | | | | |
| B24 | Have you ever told anyone about your HIV status after testing? | YES ………………………………………………….1  NO ………………………………………………….2  NOT APPLICABLE …………………………….66 | | | | | | 1  2  66 | | | | |
| **Section C: Current partnership *To be completed for those who are currently married or living with a partner.***  *‘I am going to ask you some questions about your current partnership. I am interested in both formal marriages that have taken place through a church or civil ceremony as well as traditional marriages and informal marriages where you and your husband just started living together.’* | | | | | | | | | | | | |
| C01 | For how long have you been with your current partner? | *(If < 1 year complete months otherwise complete years only)*  (years) (months) | | | | | | | | | | |
| C08 | Are you still living with your current partner?  *(By living we mean sharing meals on a regular basis)* | YES ........................................1  NO.........................................2 | | | | | 1  2 | | | | | |
| C09 | How do you rate your relationship with your current partner? | EXCELLENT ...........................1  GOOD ...................................2  NEITHER GOOD NOR BAD.....3  BAD ......................................4  TERRIBLE...............................5  REFUSED TO ANSWER ........77  DON’T KNOW .....................88 | | | | | 1  2  3  4  5  77  88 | | | | | |
| C10 | How many times has your partner ever taken money from you against your will? | NEVER ..................................1  ONCE ....................................2  2-4 TIMES .............................3  MORE THAN 4 TIMES ...........4  REFUSED TO ANSWER ........77  DON’T KNOW .....................88 | | | | | 1  2  3  4  77  88 | | | | | |
| C11 | How controlling would you say your current partner is? | VERY CONTROLLING..............1  CONTROLLING ......................2  SLIGHTLY CONTROLLING ......3  NEVER CONTROLLING ..........4  REFUSED TO ANSWER ........77  DON’T KNOW .....................88 | | | | | 1  2  3  4  77  88 | | | | | |
| C12 | How often do you use a condom when you have sex with your current partner? | EVERY TIME …………………………………….1  SOMETIMES ……………………………………2  NEVER …………………………………………….3 | | | | | 1  2  3 | | | | | |
| C13 | How often have you used a condom in the past 3 months when having sex with your current partner? | EVERY TIME …………………………………….1  SOMETIMES ……………………………………2  NEVER …………………………………………….3 | | | | | 1  2  3 | | | | | |
| C14 | Does your partner have sexual partners other than yourself? | YES........................................1  NO ........................................2  DON’T KNOW......................88 | | | | | 1  2  88 | | | | | |
| C15 | Has your current partner ever hit/kicked/hurt or threatened you? | YES........................................1  NO ........................................2  REFUSED TO ANSWER ………..77 | | | | | 1  2  77 | | | | | |
| C16 | If yes, how many times in the last 3 months? | NUMBER ………………………… | | | | |  | | | | | |
| C17 | Have you ever hit/kicked/hurt or threatened your current partner? | YES........................................1  NO ........................................2  REFUSED TO ANSWER ………..77 | | | | | 1  2  77 | | | | | |
| C18 | If yes, how many times in the last 3 months? | NUMBER ……………………. | | | | |  | | | | | |
| C19 | Has your current partner ever forced you to have sex against your will? | YES........................................1  NO ........................................2  REFUSED TO ANSWER ………..77 | | | | | 1  2  77 | | | | | |
| C20 | If yes, how many times in the last 3 months have you been forced to have sex against your will? | NUMBER ………………………… | | | | |  | | | | | |
| C21 | Have you ever forced your current partner to have sex against their will? | YES........................................1  NO ........................................2  REFUSED TO ANSWER ………..77 | | | | | 1  2  77 | | | | | |
| C22 | If yes, how many times in the last 3 months have you forced your partner to have sex against their will? | NUMBER ………………………… | | | | |  | | | | | |
| *Now I am going to ask you some questions about your recent decision to test for HIV. I do not want to know the results.* | | | | | | | | | | | | |
| C23 | Have you and your partner previously tested for HIV since being together? | I have tested | | | My partner has tested | | | | | | We have tested together | |
|  |  | YES.......1  NO........2 | | | YES.......1  NO........2  DON’T KNOW …….88 | | | | | | YES.......1  NO........2 | |
| C24 | Were you aware of your partner’s HIV status before testing this time? | YES ...............................1  NO.................................2 | | | | | | | | 1  2 | | |
| *Can you please help me to understand what the self-testing experience was like for you the last time by helping me to tick the boxes that are relevant to your experience for each stage of the process.* | | | | | | | | | | | | |
|  | When you tested, did you do the following  YES ...1 NO... 2 | At all | | By myself | | With my partner | | | With someone else | | | Other *(specify)* |
| C25a | Collection of kit | 1  2 | | 1  2 | | 1  2 | | | 1  2 | | | 1  2 |
| C25b | Pre-test counselling | 1  2 | | 1  2 | | 1  2 | | | 1  2 | | | 1  2 |
| C25c | Testing | 1  2 | | 1  2 | | 1  2 | | | 1  2 | | | 1  2 |
| C25d | Reading results | 1  2 | | 1  2 | | 1  2 | | | 1  2 | | | 1  2 |
| C25e | Post-test counselling with community counsellor | 1  2 | | 1  2 | | 1  2 | | | 1  2 | | | 1  2 |
| C26 | How did you access the test kit? | COUNSELOR-INITIATED ………………..1  CLIENT-INITIATED ………………………..2  DON’T KNOW ………………………….. 88 | | | | | | 1  2  88 | | | | |
| C27 | Did you share your results with your partner? | YES ......................................1  NO.........................................2 | | | | | | 1  2 | | | | |
| C28 | Did your partner share his/her results with you? | YES ......................................1  NO.........................................2  REFUSED TO ANSWER ........77 | | | | | | 1  2  77 | | | | |
| C29 | Who do you feel influenced you most to test? | PARTNER ..............................1  RELATIVE...............................2  FRIEND/NEIGHBOUR............3  HITTB TEAM MEMBER..........4  NO-ONE................................5 | | | | | | 1  2  3  4  5 | | | | |
| C30 | How much was your decision to test influenced by your partner? | TOTALLY ...............................1  SOMEWHAT.........................2  NOT MUCH ..........................3  NOT AT ALL ..........................4 | | | | | | 1  2  3  4 | | | | |
| C31 | How far do you feel you were forced into testing by your partner, whether you tested separately or together? | TOTALLY ...............................1  SOMEWHAT.........................2  NOT MUCH ..........................3  NOT AT ALL ..........................4 | | | | | | 1  2  3  4 | | | | |
