# Supplementary material for: Measuring sexual behaviour in Malawi: a triangulation of three data collection instruments
Source: BMC Public Health. 2018 Jun 28;18:807. doi: 10.1186/s12889-018-5717-x (PMC6022416; doi:10.1186/s12889-018-5717-x)
Supplement: Supplementary file 1 — Data tools. (ZIP 2282 kb) [file 12889_2018_5717_MOESM1_ESM.zip › LDS 01_FFQ _ENGLISH_FinalR2.docx]

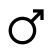

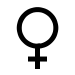

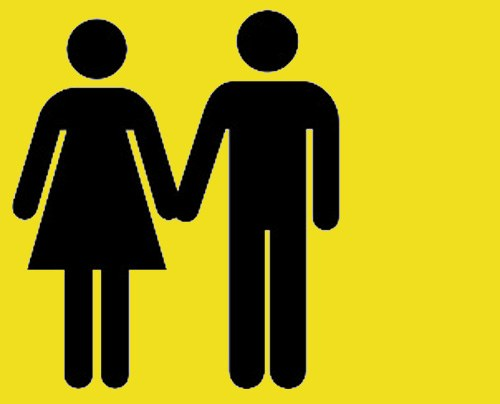

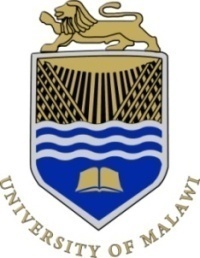

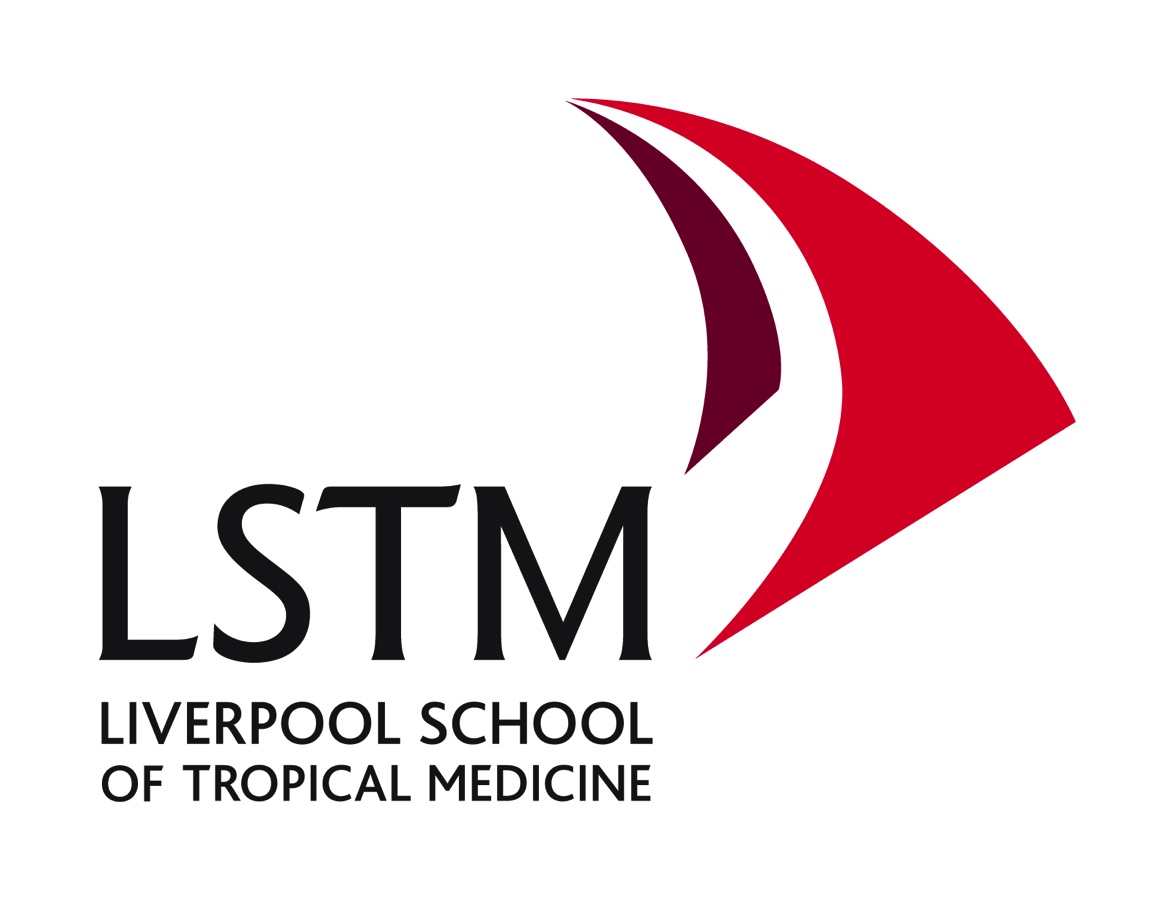

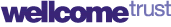

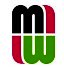


LDS //

Participant ID Complete if couple

Insert M/F

**ST IMPACTS Face to face questionnaire: LDS01**

| **Details of Interview** ***To be completed by Researcher*** | | | |
| --- | --- | --- | --- |
| Date of Interview | | Researcher ID | |
| **Section A: Demographic Details *To be completed for all participants***  *‘First I am going to ask you some questions about yourself and your life’ (Section A and B)* | | | |
| A01 | What sex are you? | MALE ………………………………………………………1  FEMALE …………………………………………………..2 | 1  2 |
| A02 | How old are you? | NUMBER …………………………… |  |
| A03 | What year were you born? | YEAR ………………………………… |  |
| A04 | What would you describe as your main religion? | CATHOLIC/LUTHERAN/PROTESTANT...1  CHRISTIANITY OTHER ……………………….2  ISLAM...................................................3  PAGANISM...........................................4  ATHEIST ..............................................5  REFUSED TO ANSWER ……….............77 | 1  2  3  4  5  77 |
| A05 | How would you describe your current marriage status? | NEVER MARRIED ………………………………………1  MARRIED OR LIVING WITH PARTNER ……….2  REMARRIED AFTER DIVORCE/DEATH ……….3  DIVORCED/SEPARATED …………………………….4  WIDOWED ……………………………………………….5 | 1  2  3  4  5 |
| A06 | How many living children do you have? | NUMBER ............................... |  |
| A07 | Of all your living children how many are currently dependent on you? | NUMBER ............................... |  |
| A08 | Of all your living children, how many are currently living with you?  ***(Enter number of children against each group)*** | BOTH MINE & PARTNER’S CHILDREN ………. 1  MY OWN CHILDREN ………………………………… 2  MY PARTNER’S CHILDREN …………………….…. 3  OTHER CHILDREN ……………………………………. 4 | 1 …………………  2 ………………….  3 …………………  4 …………………. |
| A09 | How many other dependents are living with you *(other than your partner)* excluding children? | MY RELATIVES …………………………………………1  MY PARTNER’S RELATIVES ………………………2  OTHER *(specify) ………………………………………3* | 1 ………………….  2 ………………….  3 …………………. |
| **Section B: Individual behaviour** | | | |
| B01 | How many times have you been married in your lifetime including (if relevant) your current marriage? | NUMBER ……………………………… |  |
| B02 | How many sexual partners have you had in your lifetime? | NUMBER ............................... |  |
| B03 | In the past three months how many sexual partners have you had? | NUMBER ............................... |  |
| B04 | Of your partners in the last 3 months how many were regular partners *(a regular partner is someone who you have sex with often and who provides support to meet your daily needs – they may or may not live with you*) | NUMBER ............................... |  |
| B05 | How many days is it since you last had sex? | NUMBER ................................ |  |
| B06 | With whom did you have sex most recently? | HUSBAND/ PARTNER RESIDENT…………1  REGULAR PARTNER NON-RESIDENT.....2  IRREGULAR PARTNER…………………………3  OTHER (specify)……………………………....99  REFUSED TO ANSWER……………………..77 | 1  2  3  99  77 |
| B07 | Did you use a condom last time you had sex? | YES ……………………………………………………1  NO ……………………………………………………2  REFUSED TO ANSWER …………………….77 | 1  2  77 |
| B08 | In the past 3 months how regularly have you used a condom when having sex? | EVERY TIME …………………………………….1  SOMETIMES ……………………………………2  NEVER …………………………………………….3 | 1  2  3 |

| B09 | Do you sometimes drink alcohol? | YES ……………………………………………………1  NO ……………………………………………………2  REFUSED TO ANSWER …………………….77 | 1  2  77 |
| --- | --- | --- | --- |
| B10 | In the past 3 months how often have you had drinks containing alcohol? | EVERY DAY.....................................1  AT LEAST ONCE A WEEK……………...2  AT LEAST ONCE A MONTH.............3  AT LEAST ONCE IN 3 MONTHS …….4  NEVER............................................5 | 1  2  3  4  5 |
| B11 | How many drinks do you normally have when drinking alcohol?*(One drink is defined as one cup of beer or one carton of chibuku etc.)* | NUMBER ................................ |  |
| B12 | In the last 3 months how often did you drink alcohol before having sex? | NEVER ..................................1  ONCE ....................................2  2-4 TIMES .............................3  MORE THAN 4 TIMES ...........4  REFUSED TO ANSWER ........77  DON’T KNOW .....................88 | 1  2  3  4  77  88 |
| B13 | Have you ever in your life used drugs? | YES ……………………………………………………1  NO ……………………………………………………2  REFUSED TO ANSWER …………………….77 | 1  2  77 |
| B14 | In the past 3 months how often have you used drugs e.g. marijuana? | EVERY DAY.....................................1  AT LEAST ONCE A WEEK……………...2  AT LEAST ONCE A MONTH.............3  AT LEAST ONCE IN 3 MONTHS …….4  NEVER............................................5 | 1  2  3  4  5 |
| B15 | Have you previously self-tested through HitTB? | YES ......................................1  NO.........................................2 | 1  2 |
| B16 | Other than self-testing with HitTB, have you previously tested for HIV in your lifetime? | YES ……………………………………………………1  NO ……………………………………………………2  REFUSED TO ANSWER …………………….77 | 1  2  77 |
| B17 | Other than self-testing with HitTB, how many times have you tested for HIV in your lifetime? | NUMBER …………………………………….. |  |
| B18 | Other than self-testing with HitTB, have you previously tested for HIV with a partner in your lifetime *(couples testing)?* | YES ……………………………………………………1  NO ……………………………………………………2  REFUSED TO ANSWER …………………….77 | 1  2  77 |
| B19 | Other than self-testing with HitTB, how many times have you previously tested for HIV with a partner in your lifetime? | NUMBER ……………………….. |  |
| B20 | Have you ever been persuaded to test for HIV? | YES ……………………………………………………1  NO ……………………………………………………2 | 1  2 |
| B21 | If yes, who persuaded you to test for HIV? | PARTNER ………………………………………….1  OTHER FAMILY MEMBER ………………….2  FRIEND/NEIGHBOUR ………………………..3  HEALTH WORKER …………………………….4  OTHER *(Please specify) ………………………*.99 | 1  2  3  4  99 |
| B22 | Have you ever been forced to test for HIV? | YES ……………………………………………………1  NO ……………………………………………………2 | 1  2 |
| B23 | If yes, who forced you to test for HIV? | PARTNER ………………………………………….1  OTHER FAMILY MEMBER ………………….2  FRIEND/NEIGHBOUR ………………………..3  HEALTH WORKER …………………………….4  OTHER *(Please specify) ……………………*.99 | 1  2  3  4  99 |
| B24 | Have you ever told anyone about your HIV status after testing? | YES ………………………………………………….1  NO ………………………………………………….2  NOT APPLICABLE …………………………….66 | 1  2  66 |
| B25 | If you were positive, would you tell your sexual partner? | YES ……………………………………………………1  NO ……………………………………………………2 | 1  2 |
| B26 | Has anyone ever hit/kicked/hurt or threatened you with violence? | YES ……………………………………………………1  NO ……………………………………………………2 | 1  2 |
| B27 | Have you ever hit/kicked/hurt or threatened anyone with violence? | YES ……………………………………………………1  NO ……………………………………………………2 | 1  2 |
| B28 | Have you ever been forced to have sex against your will? | YES ……………………………………………………1  NO ……………………………………………………2 | 1  2 |
| B29 | Have you ever forced someone to have sex against their will? | YES ……………………………………………………1  NO ……………………………………………………2 | 1  2 |

| **Section C: Current partnership *To be completed for those who are currently married or living with a partner.***  *‘I am going to ask you some questions about your current partnership. I am interested in both formal marriages that have taken place through a church or civil ceremony as well as traditional marriages and informal marriages where you and your husband just started living together.’* | | | | | | | | | | | | | | | | | | | | | | | | | | | | | |
| --- | --- | --- | --- | --- | --- | --- | --- | --- | --- | --- | --- | --- | --- | --- | --- | --- | --- | --- | --- | --- | --- | --- | --- | --- | --- | --- | --- | --- | --- |
| C01 | For how long have you been with your current partner? | | | | | | *(If < 1 year complete months otherwise complete years only)*  (years) (months) | | | | | | | | | | | | | | | | | | | | | | |
| C02 | How many children do you have with your current partner? | | | | | | NUMBER ............................. | | | | | | | | | | |  | | | | | | | | | | | |
| C03 | Do you plan to have children/more children with your current partner? | | | | | | YES........................................1  NO ........................................2  DON’T KNOW......................88 | | | | | | | | | | | 1  2  88 | | | | | | | | | | | |
| C04 | In which year did you marry/begin living with your current husband/wife? | | | | | | YEAR .................................  CAN’T REMEMBER .......8888 | | | | | | | | | | |  | | | | | | | | | | | |
| C05 | After you and your current partner got married, where did you live? | | | | | | HUSBAND’S VILLAGE.............1  WIFE’S VILLAGE.....................2  SOMEWHERE ELSE................3 | | | | | | | | | | | 1  2  3 | | | | | | | | | | | |
| C06 | Who owns/pays for the house you moved to after getting married? | | | | | | HUSBAND.............. ...............1  WIFE......................................2  HUSBAND’S FAMILY..............3  WIFE’S FAMILY .....................4  OTHER UNRELATED ..............5 | | | | | | | | | | | 1  2  3  4  5 | | | | | | | | | | | |
| C07 | Is this the place you are still living now? | | | | | | YES ........................................1  NO.........................................2 | | | | | | | | | | | 1  2 | | | | | | | | | | | |
| C08 | Are you still living with your current partner?  *(By living we mean sharing meals on a regular basis)* | | | | | | YES ........................................1  NO.........................................2 | | | | | | | | | | | 1  2 | | | | | | | | | | | |
| C09 | How do you rate your relationship with your current partner? | | | | | | EXCELLENT ...........................1  GOOD ...................................2  NEITHER GOOD NOR BAD.....3  BAD ......................................4  TERRIBLE...............................5  REFUSED TO ANSWER ........77  DON’T KNOW .....................88 | | | | | | | | | | | 1  2  3  4  5  77  88 | | | | | | | | | | | |
| C10 | How many times has your partner ever taken money from you against your will? | | | | | | NEVER ..................................1  ONCE ....................................2  2-4 TIMES .............................3  MORE THAN 4 TIMES ...........4  REFUSED TO ANSWER ........77  DON’T KNOW .....................88 | | | | | | | | | | | 1  2  3  4  77  88 | | | | | | | | | | | |
| C11 | How controlling would you say your current partner is? | | | | | | VERY CONTROLLING..............1  CONTROLLING ......................2  SLIGHTLY CONTROLLING ......3  NEVER CONTROLLING ..........4  REFUSED TO ANSWER ........77  DON’T KNOW .....................88 | | | | | | | | | | | 1  2  3  4  77  88 | | | | | | | | | | | |
| C12 | How often do you use a condom when you have sex with your current partner? | | | | | | EVERY TIME …………………………………….1  SOMETIMES ……………………………………2  NEVER …………………………………………….3 | | | | | | | | | | | 1  2  3 | | | | | | | | | | | |
| C13 | How often have you used a condom in the past 3 months when having sex with your current partner? | | | | | | EVERY TIME …………………………………….1  SOMETIMES ……………………………………2  NEVER …………………………………………….3 | | | | | | | | | | | 1  2  3 | | | | | | | | | | | |
| C14 | Does your partner have sexual partners other than yourself? | | | | | | YES........................................1  NO ........................................2  DON’T KNOW......................88 | | | | | | | | | | | 1  2  88 | | | | | | | | | | | |
| C15 | Has your current partner ever hit/kicked/hurt or threatened you? | | | | | | YES........................................1  NO ........................................2  REFUSED TO ANSWER ………..77 | | | | | | | | | | | 1  2  77 | | | | | | | | | | | |
| C16 | If yes, how many times in the last 3 months? | | | | | | NUMBER ………………………… | | | | | | | | | | |  | | | | | | | | | | | |
| C17 | Have you ever hit/kicked/hurt or threatened your current partner? | | | | | | YES........................................1  NO ........................................2  REFUSED TO ANSWER ………..77 | | | | | | | | | | | 1  2  77 | | | | | | | | | | | |
| C18 | If yes, how many times in the last 3 months? | | | | | | NUMBER ……………………. | | | | | | | | | | |  | | | | | | | | | | | |
| C19 | Has your current partner ever forced you to have sex against your will? | | | | | | YES........................................1  NO ........................................2  REFUSED TO ANSWER ………..77 | | | | | | | | | | | 1  2  77 | | | | | | | | | | | |
| C20 | If yes, how many times in the last 3 months have you been forced to have sex against your will? | | | | | | NUMBER ………………………… | | | | | | | | | | |  | | | | | | | | | | | |
| C21 | Have you ever forced your current partner to have sex against their will? | | | | | | YES........................................1  NO ........................................2  REFUSED TO ANSWER ………..77 | | | | | | | | | | | 1  2  77 | | | | | | | | | | | |
| C22 | If yes, how many times in the last 3 months have you forced your partner to have sex against their will? | | | | | | NUMBER ………………………… | | | | | | | | | | |  | | | | | | | | | | | |
| *Now I am going to ask you some questions about your recent decision to test for HIV. I do not want to know the results.* | | | | | | | | | | | | | | | | | | | | | | | | | | | | | |
| C23 | Have you and your partner previously tested for HIV since being together? | | | | | | I have tested | | | | | | | My partner has tested | | | | | | | | | | | We have tested together | | | | |
|  |  |  |  |  |  |  | YES.......1  NO........2 | | | | | | | YES.......1  NO........2  DON’T KNOW …….88 | | | | | | | | | | | YES.......1  NO........2 | | | | |
| C24 | Were you aware of your partner’s HIV status before testing this time? | | | | | | YES ...............................1  NO.................................2 | | | | | | | | | | | | | | 1  2 | | | | | | | | |
| *Can you please help me to understand what the self-testing experience was like for you the last time by helping me to tick the boxes that are relevant to your experience for each stage of the process* | | | | | | | | | | | | | | | | | | | | | | | | | | | | | |
|  | When you tested, did you do the following  YES ...1 NO... 2 | | | | | | At all | | | | By myself | | | | | With my partner | | | | | | | With someone else | | | | | Other *(specify)* | |
| C25a | Collection of kit | | | | | | 1  2 | | | | 1  2 | | | | | 1  2 | | | | | | | 1  2 | | | | | 1  2 | |
| C25b | Pre-test counselling | | | | | | 1  2 | | | | 1  2 | | | | | 1  2 | | | | | | | 1  2 | | | | | 1  2 | |
| C25c | Testing | | | | | | 1  2 | | | | 1  2 | | | | | 1  2 | | | | | | | 1  2 | | | | | 1  2 | |
| C25d | Reading results | | | | | | 1  2 | | | | 1  2 | | | | | 1  2 | | | | | | | 1  2 | | | | | 1  2 | |
| C25e | Post-test counselling with community counsellor | | | | | | 1  2 | | | | 1  2 | | | | | 1  2 | | | | | | | 1  2 | | | | | 1  2 | |
| C26 | How did you access the test kit? | | | | | | COUNSELOR-INITIATED ………………..1  CLIENT-INITIATED ………………………..2  DON’T KNOW ………………………….. 88 | | | | | | | | | | | | 1  2  88 | | | | | | | | | | |
| C27 | Did you share your results with your partner? | | | | | | YES .......................................1  NO.........................................2 | | | | | | | | | | | | 1  2 | | | | | | | | | | |
| C28 | Did your partner share his/her results with you? | | | | | | YES ........................................1  NO.........................................2  REFUSED TO ANSWER ........77 | | | | | | | | | | | | 1  2  77 | | | | | | | | | | |
| C29 | Who do you feel influenced you most to test? | | | | | | PARTNER ..............................1  RELATIVE...............................2  FRIEND/NEIGHBOUR............3  HITTB TEAM MEMBER..........4  NO-ONE................................5 | | | | | | | | | | | | 1  2  3  4  5 | | | | | | | | | | |
| C30 | How much was your decision to test influenced by your partner? | | | | | | TOTALLY ...............................1  SOMEWHAT.........................2  NOT MUCH ..........................3  NOT AT ALL ..........................4 | | | | | | | | | | | | 1  2  3  4 | | | | | | | | | | |
| C31 | How far do you feel you were forced into testing by your partner, whether you tested separately or together? | | | | | | TOTALLY ...............................1  SOMEWHAT.........................2  NOT MUCH ..........................3  NOT AT ALL ..........................4 | | | | | | | | | | | | 1  2  3  4 | | | | | | | | | | |
| *(If respondent tested with partner)* How far do you agree or disagree with the following statements | | | | | | | | | | | | | | | | | | | | | | | | | | | | | |
|  | |  | | Strongly agree | | | | Agree | | | | Neither agree nor disagree | | | | | | | | Strongly disagree | | | | | | Strongly disagree | | | |
| C32a | | I am happy that I tested with my partner | | 1 | | | | 2 | | | | 3 | | | | | | | | 4 | | | | | | 5 | | | |
| C32b | | I regret testing with my partner | | 1 | | | | 2 | | | | 3 | | | | | | | | 4 | | | | | | 5 | | | |
| C32c | | I feel that I was forced to test by my partner | | 1 | | | | 2 | | | | 3 | | | | | | | | 4 | | | | | | 5 | | | |
| C32d | | I feel that I forced my partner to test | | 1 | | | | 2 | | | | 3 | | | | | | | | 4 | | | | | | 5 | | | |
| C32e | | I would choose to test with my partner next time I take an HIV test | | 1 | | | | 2 | | | | 3 | | | | | | | | 4 | | | | | | 5 | | | |
| C32f | | I feel my relationship is stronger after testing as a couple | | 1 | | | | 2 | | | | 3 | | | | | | | | 4 | | | | | | 5 | | | |
| **Section D: Household dynamics** *To be completed by those living with and without partners* | | | | | | | | | | | | | | | | | | | | | | | | | | | | | |
| ‘For each of the individuals named (where relevant), can you tell me who provides most of their food and daily needs in your household currently?’ | | | | | | | | | | | | | | | | | | | | | | | | | | | | | |
|  |  | | Husband | | | Wife | | | | Both husband & wife | | | Other family member | | | | Another boy/  girlfriend | | | | | | | Not applicable | | | | | Other *(specify)* |
| D01a | Husband | | 1 | | | 2 | | | | 3 | | | 4 | | | | 5 | | | | | | | 66 | | | | | 99 |
| D01b | Wife | | 1 | | | 2 | | | | 3 | | | 4 | | | | 5 | | | | | | | 66 | | | | | 99 |
| D01c | Male children | | 1 | | | 2 | | | | 3 | | | 4 | | | | 5 | | | | | | | 66 | | | | | 99 |
| D01d | Female children | | 1 | | | 2 | | | | 3 | | | 4 | | | | 5 | | | | | | | 66 | | | | | 99 |
| D01e | Other dependents | | 1 | | | 2 | | | | 3 | | | 4 | | | | 5 | | | | | | | 66 | | | | | 99 |
| ‘In your household who generally makes the following decisions currently?’ | | | | | | | | | | | | | | | | | | | | | | | | | | | | | |
|  |  | | | | Husband | | | | Wife | | | | | | Both husband & wife | | | | | | | Other family member | | | | | Don’t know | | |
| D02a | If you need to buy clothes for the family | | | | 1 | | | | 2 | | | | | | 3 | | | | | | | 4 | | | | | 88 | | |
| D02b | If you need to buy cooking utensils | | | | 1 | | | | 2 | | | | | | 3 | | | | | | | 4 | | | | | 88 | | |
| D02c | If you need to buy large household items | | | | 1 | | | | 2 | | | | | | 3 | | | | | | | 4 | | | | | 88 | | |
| D02d | What foods should be cooked each day | | | | 1 | | | | 2 | | | | | | 3 | | | | | | | 4 | | | | | 88 | | |
| D02e | Whether a child is sick enough to go for treatment | | | | 1 | | | | 2 | | | | | | 3 | | | | | | | 4 | | | | | 88 | | |
| D02f | Whether the husband is sick enough to go for treatment | | | | 1 | | | | 2 | | | | | | 3 | | | | | | | 4 | | | | | 88 | | |
| D02g | Whether the wife is sick enough to go for treatment | | | | 1 | | | | 2 | | | | | | 3 | | | | | | | 4 | | | | | 88 | | |
| D02h | Where the husband should work | | | | 1 | | | | 2 | | | | | | 3 | | | | | | | 4 | | | | | 88 | | |
| D02i | Whether the wife should work | | | | 1 | | | | 2 | | | | | | 3 | | | | | | | 4 | | | | | 88 | | |
| D02j | Where the children should be schooled | | | | 1 | | | | 2 | | | | | | 3 | | | | | | | 4 | | | | | 88 | | |

| **Section E: Previous partnerships *Only complete this section of the questionnaire if response to B02 is greater than 1. Complete for up to 4 previous partners using the male/female picture to outline a key characteristic of each partner and use this to remind the participant of the partner they should be thinking about. Complete all questions for partner #1 before continuing with partner #2 and so on* …**  ‘*I would like to ask you a few questions about your relationship with each of your previous husbands/wives (i.e. not your current husband/wife), beginning with your first husband/wif’e. (Use the year book tool to clearly define year and age when the marriage occurred)* | | | | | | |
| --- | --- | --- | --- | --- | --- | --- |
| **Question No.** | **Question** | **Response** | **Partner #1** | **Partner #2** | **Partner #3** | **Partner #4** |
| E01 | In which year did you marry/start living with this partner? | YEAR ...................................... |  |  |  |  |
| E02 | How old were you when you married/started living with this partner? | YEARS ...................................... |  |  |  |  |
| E03 | Was this partner older or younger than you when you got married? | OLDER .....................................1  YOUNGER................................2  ABOUT THE SAME AGE ...........3  DON’T KNOW ........................88 | 1  2  3  88 | 1  2  3  88 | 1  2  3  88 | 1  2  3  88 |
| E04 | What type of marriage was this?  *Probe for main type of marriage and select one category only for each partner* | LEGAL.......................................1  RELIGIOUS................................2  TRADITIONAL PATRILINEAL WITH BRIDEPRICE ...................3  TRADITIONAL MATRILINEAL ….4  INFORMAL MARRIAGE …….……5 | 1  2  3  4  5 | 1  2  3  4  5 | 1  2  3  4  5 | 1  2  3  4  5 |
| E05 | How many children did you have with this husband/wife? | NUMBER ……………………….. |  |  |  |  |
| E06 | How often did you use condoms when you had sex with this partner? | EVERY TIME ……………………….1  SOMETIMES ………………………2  NEVER ……………………………….3 | 1  2  3 | 1  2  3 | 1  2  3 | 1  2  3 |
| E07 | Did you ever test for HIV during this marriage? | YES ..........................................1  NO............................................2 | 1  2 | 1  2 | 1  2 | 1  2 |
| E08 | Did you ever test together as a couple during this marriage? | YES ..........................................1  NO............................................2 | 1  2 | 1  2 | 1  2 | 1  2 |
| E09 | Did you share your HIV test results with each other? | YES ..........................................1  NO............................................2 | 1  2 | 1  2 | 1  2 | 1  2 |
| E10 | Did you ever test positive in this relationship? | YES ..........................................1  NO............................................2 | 1  2 | 1  2 | 1  2 | 1  2 |
| E11 | Did your partner ever test positive in this relationship? | YES ..........................................1  NO............................................2  Don’t know............................88 | 1  2  88 | 1  2  88 | 1  2  88 | 1  2  88 |
| E12 | In which year did this marriage end? | YEAR .........................................  CAN’T REMEMBER ............8888 |  |  |  |  |
| E13 | How did this marriage end? | DIVORCED ..............1  SEPARATED............2  WIDOWED.............3 | 1  2  3 | 1  2  3 | 1  2  3 | 1  2  3 |
| E14 | If divorced or separated from partner, what were the reasons for this?  *(Select all reasons cited by respondent)* | HUSBAND/WIFE UNFAITHFUL.1  HUSBAND DID NOT PROVIDE...2 HUSBAND MARRIED OTHER WIFE ........................................3  WIFE DID NOT FULFILL HER DOMESTIC DUTIES ..................4  HUSBAND DID NOT FULFILL HIS DOMESTIC DUTIES...................5  I WAS UNFAITHFUL .................6  LACK OF LOVE ........................7  WORK/MIGRATION .................8  PARTNER WAS VIOLENT ..........9  OTHER *(specify).......................*99 | 1  2  3  4  5  6  7  8  9  99 | 1  2  3  4  5  6  7  8  9  99 | 1  2  3  4  5  6  7  8  9  99 | 1  2  3  4  5  6  7  8  9  99 |
| E15 | If divorced or separated from partner, what was the main reason for the divorce/separation?  *(Select one reason only)* | HUSBAND/WIFE UNFAITHFUL.1  HUSBAND DID NOT PROVIDE...2 HUSBAND MARRIED OTHER WIFE ........................................3  WIFE DID NOT FULFILL HER DOMESTIC DUTIES ..................4  HUSBAND DID NOT FULFILL HIS DOMESTIC DUTIES...................5  I WAS UNFAITHFUL .................6  LACK OF LOVE ........................7  WORK/MIGRATION .................8  PARTNER WAS VIOLENT ..........9  OTHER *(specify).......................*99 | 1  2  3  4  5  6  7  8  9  99 | 1  2  3  4  5  6  7  8  9  99 | 1  2  3  4  5  6  7  8  9  99 | 1  2  3  4  5  6  7  8  9  99 |
| E16 | Did this partner ever force you to have sex against your will? | YES ..........................................1  NO............................................2 | 1  2 | 1  2 | 1  2 | 1  2 |
| E17 | Did you ever force this partner to have sex with you against their will? | YES ..........................................1  NO............................................2 | 1  2 | 1  2 | 1  2 | 1  2 |
| *Thank you for taking the time to answer these questions, all your answers will be kept anonymous and confidential. Do you have any questions you would like to ask me?* | | | | | | |
